# ErbB4 promotes malignant peripheral nerve sheath tumor pathogenesis via Ras-independent mechanisms

**DOI:** 10.1186/s12964-019-0388-5

**Published:** 2019-07-10

**Authors:** Jody Fromm Longo, Stephanie N. Brosius, Laurel Black, Stuart H. Worley, Robert C. Wilson, Kevin A. Roth, Steven L. Carroll

**Affiliations:** 1 Department of Pathology (SNB, KAR) and the Medical Scientist Training Program (SNB), University of Alabama at Birmingham, Birmingham, AL 35294-0017 USA; 2 Department of Pathology and Laboratory Medicine (JFL, LB, RCW, SJW, SLC), Medical University of South Carolina, 171 Ashley Avenue, MSC 908, Charleston, SC 29425-9080 USA; 3 Present address: Department of Pediatrics at The Children’s Hospital of Philadelphia, Philadelphia, PA USA; 4 Present address: Department of Pathology and Cell Biology, Vagelos College of Physicians and Surgeons, Columbia University, New York City, NY USA

**Keywords:** Neurofibromatosis type 1, Schwann cell, erbB kinases, Cytoplasmic signaling pathways

## Abstract

**Background:**

We have found that erbB receptor tyrosine kinases drive Ras hyperactivation and growth in *NF1*-null malignant peripheral nerve sheath tumors (MPNSTs). However, MPNSTs variably express multiple erbB receptors with distinct functional characteristics and it is not clear which of these receptors drive MPNST pathogenesis. Here, we test the hypothesis that altered erbB4 expression promotes MPNST pathogenesis by uniquely activating key cytoplasmic signaling cascades.

**Methods:**

ErbB4 expression was assessed using immunohistochemistry, immunocytochemistry, immunoblotting and real-time PCR. To define erbB4 functions, we generated mice that develop MPNSTs with floxed *Erbb4* alleles (P_0_-GGFβ3;*Trp53*^*+/−*^*;Erbb4*^*flox/flox*^ mice) and ablated *Erbb4* in these tumors. MPNST cell proliferation and survival was assessed using ^3^H-thymidine incorporation, MTT assays, Real-Time Glo and cell count assays. Control and *Erbb4*-null MPNST cells were orthotopically xenografted in immunodeficient mice and the growth, proliferation (Ki67 labeling), apoptosis (TUNEL labeling) and angiogenesis of these grafts was analyzed. Antibody arrays querying cytoplasmic kinases were used to identify erbB4-responsive kinases. Pharmacologic or genetic inhibition was used to identify erbB4-responsive kinases that drive proliferation.

**Results:**

Aberrant erbB4 expression was evident in 25/30 surgically resected human MPNSTs and in MPNSTs from genetically engineered mouse models (P_0_-GGFβ3 and P_0_-GGFβ3;*Trp53*^*+/−*^ mice); multiple erbB4 splice variants that differ in their ability to activate PI3 kinase and nuclear signaling were present in MPNST-derived cell lines. *Erbb4*-null MPNST cells demonstrated decreased proliferation and survival and altered morphology relative to non-ablated controls. Orthotopic allografts of *Erbb4*-null cells were significantly smaller than controls, with reduced proliferation, survival and vascularization. *ERBB4* knockdown in human MPNST cells similarly inhibited DNA synthesis and viability. Although we have previously shown that broad-spectrum erbB inhibitors inhibit Ras activation, *Erbb4* ablation did not affect Ras activation, suggesting that erbB4 drives neoplasia via non-Ras dependent pathways. An analysis of 43 candidate kinases identified multiple NRG1β-responsive and erbB4-dependent signaling cascades including the PI3K, WNK1, STAT3, STAT5 and phospholipase-Cγ pathways. Although WNK1 inhibition did not alter proliferation, inhibition of STAT3, STAT5 and phospholipase-Cγ markedly reduced proliferation.

**Conclusions:**

ErbB4 promotes MPNST growth by activating key non-Ras dependent signaling cascades including the STAT3, STAT5 and phospholipase-Cγ pathways. ErbB4 and its effector pathways are thus potentially useful therapeutic targets in MPNSTs.

**Electronic supplementary material:**

The online version of this article (10.1186/s12964-019-0388-5) contains supplementary material, which is available to authorized users.

## Background

Malignant peripheral nerve sheath tumors (MPNSTs) are highly aggressive spindle cell neoplasms derived from the Schwann cell lineage [1]. These neoplasms are the most common malignancy occurring in patients with neurofibromatosis type 1 (NF1) [2]; they also occur sporadically in the general population and at sites of previous radiation therapy. There is an ongoing controversy as to whether NF1-associated, sporadic or radiation-induced MPNSTs have a worse prognosis [3–7]. However, it is generally agreed that in all of these clinical settings, MPNSTs have a poor prognosis, with several academic centers reporting 5-year disease-free survival rates of 34–60% [4, 6–12]. In large part, these poor outcomes reflect the fact that surgical resection is currently the only effective means of treating MPNSTs and achieving a complete surgical resection is often impossible. Developing effective new chemotherapeutic regimens is thus essential if we are to improve the survival of patients with these aggressive neoplasms.

Loss-of-function mutations of the *NF1* tumor suppressor gene, which encodes the Ras inhibitor neurofibromin, are present in all NF1-associated MPNSTs and a major subset of sporadic and radiation-induced MPNSTs [13, 14]. In the absence of neurofibromin, Ras activation is unopposed, resulting in Ras hyperactivation. Given this, it was reasonable to expect that agents targeting Ras or Ras-regulated cytoplasmic signaling cascades would be effective against MPNSTs. However, attempts to treat MPNSTs in this manner have thus far been unsuccessful. This reflects the fact that multiple Ras proteins are hyperactivated in MPNSTs [15] and that the key Ras-regulated signaling pathways in these tumors are poorly understood. This led us to hypothesize that an alternative approach, namely targeting the upstream proteins that drive Ras hyperactivation in *NF1*-null MPNSTs would be effective against MPNSTs. Prior to testing this hypothesis, though, we must identify the key Ras activating proteins in MPNSTs. Several lines of evidence suggest that one or more members of the erbB family of receptor tyrosine kinases (RTKs) fulfill this role in MPNSTs. We and others have shown that MPNSTs variably express erbB1 (the EGF receptor), erbB2, erbB3 and erbB4 [16]. These erbB kinases are constitutively activated in MPNST cells [16] and promote their proliferation [16] and migration [17]. Further, when we overexpressed an erbB3/erbB4-specific ligand [the neuregulin-1 (NRG1) isoform glial growth factor-β3 (GGFβ3)] in the Schwann cells of transgenic mice (P_0_-GGFβ3 mice), these animals developed neurofibromas [18] that transformed into MPNSTs in vivo [18, 19] with genomic abnormalities analogous to those seen in human MPNSTs [18]. Our subsequent genetic complementation experiments showed that NRG1 promotes MPNST pathogenesis by activating erbB3 and erbB4-mediated signaling cascades that are dysregulated by neurofibromin loss [20].

These observations present a conundrum, though—NRG1 activates both erbB3 and erbB4 and these receptors are often co-expressed in MPNSTs. Consequently, it is not clear which of them drives MPNST pathogenesis. This distinction has important mechanistic and therapeutic implications. NRG1 binds to erbB4 with an affinity an order of magnitude greater than that of erbB3 and, when activated, erbB4 has greater tyrosine kinase activity [21]. ErbB4 also responds to several ligands (NRG3, NRG4, betacellulin, epiregulin, heparin-binding EGF) that do not activate erbB3. As their intracellular domains contain distinct docking sites for cytoplasmic signaling molecules, the downstream signaling pathways activated by erbB4 differ from those regulated by erbB3 [21, 22]. Further, erbB4, unlike other erbB kinases, can be proteolytically cleaved post-activation [23, 24], releasing a cytoplasmic fragment that functions as a transcriptional regulator [25]. It is thus likely that if erbB4 promotes MPNST pathogenesis, it does so via mechanisms different from erbB3. If erbB4 promotes MPNST pathogenesis, it would also represent a potential target for new therapeutic agents such as the monoclonal anti-erbB4 antibodies that are currently in development. Consequently, here we test the hypothesis that erbB4 promotes MPNST pathogenesis and that it does so by activating cytoplasmic signaling cascades distinct from those regulated by erbB3.

## Methods

### Reagents and antibodies

A mouse anti-GAPDH monoclonal antibody (clone 6C5) was purchased from Fitzgerald Antibodies and Antigens (Concord, MA). Rabbit anti-erbB4 antibodies were from Santa Cruz (sc-284) and Abcam (ab35374; Cambridge, MA). Rabbit anti-actinin (# 6487) and anti-S100β antibodies were purchased from Cell Signaling and Dako (Carpinteria, CA), respectively. Nestin (clone rat-401), pan-Ras (clone Ras-10), and erbB4 (HFR1; #05–1133) mouse monoclonal antibodies were from Millipore (Billerica, MA). Anti-Ki67 and anti-CD31 antibodies were obtained from Abcam (ab15580 and ab23364). Actin Green-488 ready probe, and Alexa Fluor 568 were purchased from Invitrogen; Cy3-, fluorescein isothiocyanate (FITC)- and horseradish peroxidase-conjugated secondary antibodies were from Jackson Immunoresearch (West Grove, PA). IRDye 680RD and IRDye800CW secondary antibodies were from LI-COR (Lincoln, NE). Cy3 Tyramide Signal Amplification kits were from Perkin Elmer (Waltham, MA), while Immpress Polymer Detection Reagents were purchased from Vector Laboratories (Burlingame, CA). The PLCγ inhibitor U73122 (#112648–68-7) was from Selleckchem. Stat5 (CAS 285986–31-4; #573108) and Stat3 (5, 15-DPP; #D4071) inhibitors were from Sigma.

### Human MPNST specimens and lines

Paraffin blocks of surgically resected human MPNSTs were from the files of the UAB Department of Pathology. We have previously described the sources of our human MPNST cell lines [16, 17, 26, 27]. MPNST cells were maintained in Dulbecco’s modification of Eagle’s medium (DMEM) supplemented with 10% fetal calf serum, 10 μg/mL streptomycin and 10 IU/mL penicillin (DMEM10). Cell line identities were validated by short tandem repeat analyses as recommended by ATCC Technical Bulletin 8. The morphology and doubling times of these cultures were monitored and cells were tested at regular intervals by PCR for *Mycoplasma*.

### Immunoblotting and immunohistochemistry

Cells were lysed and immunoblotted using our previously described methodology [20]. Immunoreactive species were identified using enhanced chemiluminescence (Pierce) or Licor IRDye secondaries. We have found that detection of erbB4 is highly dependent on lysis conditions; for details and a comparison of erbB4 detection under different lysis conditions, see Additional file 1: Figure S1. Activated Ras was pulled down using the Raf1-Ras binding domain (ThermoFisher; Waltham, MA) and detected with a pan-Ras antibody as previously described [20]. Nestin, S100β, and Ki67 immunohistochemistry, TUNEL labeling and determination of Ki67 and TUNEL labeling indices was performed per our previously described methodologies [20]. To quantify vascular densities, 3–4 independent images of CD31-immunostained sections from each graft (Cre-ablated and control) of UBI-1 and UBI-2 cells were analyzed with ImageJ using the “automated counting of single-color image” function.

### RNA interference

A pool of four siRNAs targeting erbB4 (L-003128-00-005) and a non-targeting control pool (D-001810-10-05) were purchased from Thermo Scientific (Waltham, MA), while WNK1 (Sigma TRCN0000000919, TRCN0000219718) and erbB4 shRNAs (TRCN00000001410, TRCN00000001411) were obtained from the Hollings Cancer Center shRNA Shared Resource. Cells plated in DMEM-10 (200,000 cells/well in 6 well plates) were transfected with siRNAs using X-TREME Gene siRNA transfection reagent (Roche, Indianapolis, IN; 5:2 transfection reagent:siRNA). Cells were transduced with shRNAs using our previously described methodology [15].

### Quantitative PCR (qPCR)

1000 ng of total RNA isolated using Trizol (Life Technologies) was converted to cDNA using a SuperScript Vilo RT Kit (Invitrogen). 5–20 ng of cDNA was used for qPCR with TaqMan primer sets following the manufacturers’ protocol. The TaqMan primer sets used were: pan-erbB4: Hs00171783_m1 (spans exons 12–13, detects all isoforms), Hs00955522_m1 (spans exons 26–27; CYT1 specific), Hs00955509_m1 (spans exons 14–15; JMb specific), Hs00955511_m1 (spans exons 16–17; JMa specific), a custom set for CYT2 (spans exons 25–27; 5′ primer: 5′-CAACATCCCACCTCCCATCTATAC-3′, 3′ primer: 5′ACACTCCTTGTTCAGCAGCAAA-3′, probe a: 5′AATTGACTCGAATAGGAACCAGTTTGTATACCGAGAT-3′) and GAPDH (Hs99999905_m1, Mm99999915_g1).

### In vitro proliferation and survival assays

Tritiated thymidine incorporation assays were performed as previously described [26] with cells plated at a density of 20,000 cells per well in 48 well plates. For Real-Time Glo and MTT assays, 4,000 or 20,000 log phase cells per well were plated in 96 or 48 well plates, respectively, and assays performed following the manufacturers’ instructions. For total cell count proliferation assays the Celigo Imaging Cytometer was used in conjunction with DAPI nuclear total cell stain and propidium iodine staining. Cells were imaged every other day for five to 7 days.

### Genetically engineered mouse models

Mice were maintained according to the *NIH Guide for the Care and Use of Laboratory Animals* (Eighth Edition). Standard cages were used to house mice, with food and water available ad libitum*.*

We have previously described the generation and characterization of P_0_-GGFβ3;*Trp53*^*+/−*^ mice [20]. *Erbb4*^−/−^; α-MHC-*Erbb4* mice [28] were provided by Dr. Andres Buonanno. Mice with exon 2 of the *Erbb4* gene flanked by loxP sites (*Erbb4*^*flox/flox*^ mice) were obtained from Dr. Kent Lloyd [29]. P_0_-GGFβ3;*Trp53*^*+/−*^ mice were mated to *Erbb4*^*flox/flox*^ mice and the resulting progeny then mated to each other to generate P_0_-GGFβ3;*Trp53*^*+/−*^*;Erbb4*^*flox/flox*^ mice. Offspring were screened via PCR using previously described primers for the P_0_-GGFβ3 transgene, *Trp53* null alleles [19, 20], *Erbb4*^flox^ and *Erbb4* wild-type alleles [30].

### Diagnosis of mouse tumors

Mice were examined daily for our previously described indicators of tumor development [20]; complete necropsies were performed on mice with suspected tumors and early passage cultures prepared from tumors per our previously established methods [18, 20]. Tumor diagnoses were performed following World Health Organization (WHO) diagnostic criteria as we previously described [20].

### *Erbb4* ablation with adenoviral vectors

Mouse MPNST cells were plated (100,000 cells/mL) in DMEM10. The next morning, cultures were rinsed with PBS and infected with Ad5CMVCre-eGFP or Ad5-eGFP (Gene Transfer Vector Core, University of Iowa; Iowa City, IA) in 10 mL DMEM (MOI 100) for 8 h; 10 mL DMEM10 was then added. 24–48 h post-transfection, GFP-positive cells were sorted on a BD Biosciences FACS Aria machine using FACS Diva software (Franklin Lakes, NH). *Erbb4* deletion was assessed using previously described primers [31] which generate a 250 base pair band from recombined *ErbB4*^*flox*^ alleles and a 350 base pair band from non-recombined alleles.

### Orthotopic allografts

48 h after transduction with Ad5CMVCre-eGFP or Ad5-eGFP and FACS sorting, 50,000 GFP-positive MPNST cells were orthotopically allografted into the sciatic nerves of Hsd: Athymic Nude-Foxn1^nu^ mice (Harlan Laboratories; Indianapolis, IN) per our previously published protocol [32].

### Antibody arrays

The phosphorylation of 43 kinases and two related proteins was assessed using Proteome Profiler Phospho-Kinase Arrays (#ARY003B; R&D Systems, Minneapolis, MN). MPNST cells were serum starved overnight and then stimulated with 10 nM NRG1β for 5 min. Cells were lysed and arrays processed and developed per the manufacturer’s recommendations. Signals were quantified using the Protein Array Analyzer Plug In for FIJI.

### Differentially expressed genes and RNA sequencing

Total RNA was isolated using standard Trizol based methods from FACS-sorted UBI1, 2, and 3 cells approximately 2 days after infection with Ad5CMVCre-eGFP or Ad5-eGFP. RNA integrity was verified on an Agilent 2200 TapeStation (Agilent Technologies, Palo Alto, CA); samples with RINs ≥8 were used for sequencing. RNA-Seq libraries were prepared from total RNA (100–200 ng) using the TruSeq RNA Sample Prep Kit per the manufacturer’s protocol (Illumina, San Diego, CA). Libraries were clustered at a concentration that ensured at least 50 million reads per sample on the cBot as described by the manufacturer (Illumina, San Diego, CA). Clustered RNA-seq libraries were then sequenced using Version 4 with 1X50 cycles on an Illumina HiSeq2500. Demultiplexing was performed utilizing bcl2fastq-1.8.4 to generate Fastq files. RNA from three biological replicates was sequenced. Sequencing reads (single end reads, 50 million depth) were aligned using the DNAStar software. Partek and DNAStar were used to identify statistically significant expression changes of at least 1.5-fold up or down compared to controls. The analysis for this paper was generated using Partek® software (Ver 7.0; Partek Inc., St. Louis, MO, USA). Gene ontology analysis of statistically significant genes affected by erbB4 loss were analyzed with a ranked gene list using Panther’s over-represented analysis tools.

## Results

### Aberrant expression of erbB4 is evident in MPNSTs

**Fig. 1:**
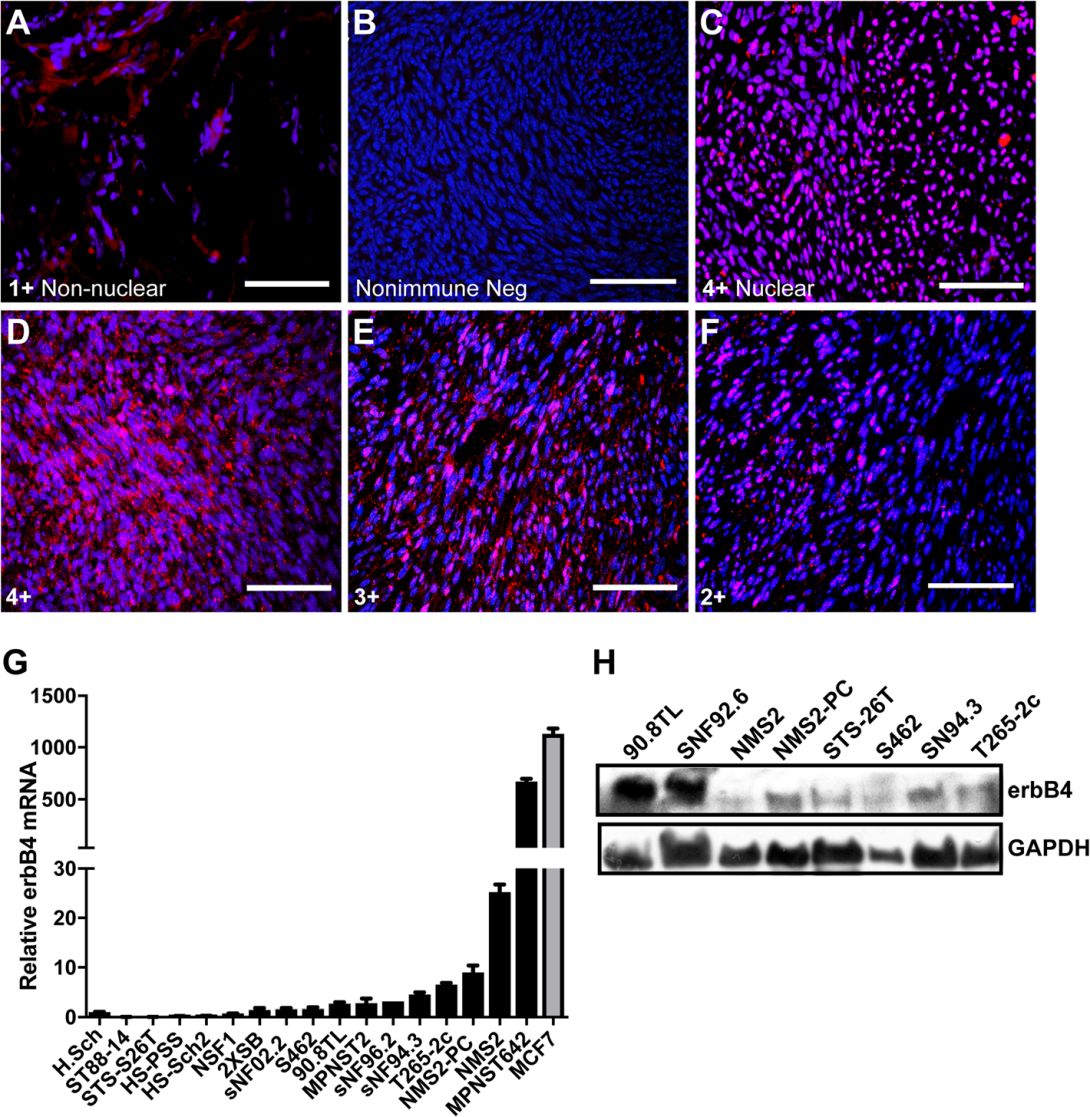
ErbB4 is commonly expressed in human MPNSTs and MPNST-derived cell lines. **a**-**f** Representative images of erbB4 immunostaining of FFPE sections of human MPNSTs demonstrates different grades of erbB4 staining compared to isotype matched negative control **b**. The erbB4 grading is represented numerically on a scale between 1+ to 4+. A subset of erbB4 positive tumor samples displayed prominent nuclear staining (**c**-**f**) and others displayed exclusive non-nuclear membranous staining (**a**). Red channel (Alexa 568, erbB4). Blue channel (Hoechst, nuclei). Scale bar represents 100um. **g** Real time PCR analysis showing relative mRNA expression of erbB4 mRNA using non-isoform discriminatory PCR primers; mRNA levels are shown relative to human Schwann cells. GAPDH mRNA was used as the loading control for normalization. **h** ErbB4 protein expression of by immunoblot analysis of whole cell lysates derived from human MPNST derived cell lines. GAPDH was used as a loading control

Our previous analyses of two MPNST cell lines and six tumor samples indicated that erbB4 was variably expressed in these neoplasms [16]. To determine how commonly erbB4 is expressed in MPNSTs in vivo and examine its distribution in these tumors, we immunostained thirty surgically resected human MPNSTs (see Additional file 1: Table S1 for the demographics and NF1 status of these patients). Twenty-five tumors (83%) were positive for erbB4 and demonstrated prominent membranous (Fig. 1a, erbB4 stain; b, control non-immune IgG stain) and/or nuclear (Fig. 1c) immunoreactivity. The membranous staining was often punctate, consistent with our previous observations in MPNST cells [17]; this is also consistent with the fact that erbB4 functions as a microenvironment sensor at the plasma membrane but can also get proteolytically cleaved and enter the nucleus to function as a transcriptional co-activator. Intratumoral variability was evident, with both erbB4-positive and –negative tumor cells present in the same neoplasm. Upon scoring the number of erbB4 positive tumor cells in each MPNST, we found that in most (16/25) tumors, the majority [> 75% (4+; Fig. 1d) or 50–75% (3+; Fig. 1e)] of the tumor cells were erbB4 immunoreactive. In another seven tumors, erbB4 staining was less widely distributed, with 25–50% of the tumor cells being erbB4 positive (2+; Fig. 1f). The remaining two erbB4-positive MPNSTs contained < 10–25% erbB4-immunoreactive cells (data not shown).

To compare erbB4 expression in MPNST cells to that in non-neoplastic human Schwann cells, we performed real time PCR quantification of *ERBB4* mRNA levels in 16 MPNST cell lines (Fig. 1g), with MCF7 cells serving as a positive high expressing control. Eleven lines had *ERBB4* expression greater than that in Schwann cells, with the highest levels observed in MPNST642, NMS2, NMS2-PC and T265-2c cells, and intermediate expression levels in 2XSB, sNF02.2, S462, 90-8TL, MPNST2, sNF96.2, and sNF94.3 cells. Four lines (ST88–14, STS-26 T, HS-PSS, Hs-Sch2) had *ERBB4* expression that was slightly lower than Schwann cells. A comparison of erbB4 protein and mRNA expression in a selected subset of MPNST lines showed that all of the lines that expressed *ERBB4* mRNA also had detectable erbB4 protein (Fig. 1h). However, *ERBB4* mRNA levels were not necessarily predictive of erbB4 protein levels, suggesting that post-translational factors regulate erbB4 protein levels in MPNST cells.

Alternative splicing of *ERBB4* mRNAs results in the production of functionally distinct protein variants that contain different juxtamembrane domains (JMa, JMb variants) and either include or lack a sequence in the autophosphorylation domain of the receptor (CYT1 and CYT2 variants, respectively). JMa variants, unlike JMb isoforms, can undergo proteolytic cleavage to release a 120 kDa fragment that acts as a transcriptional regulator. The CYT1 sequence contains a p85 docking site that allows erbB4-mediated activation of the PI3 kinase/Akt signaling cascade. To determine which *ERBB4* splice variants are predominantly expressed in MPNST cells, we performed real-time PCR with isoform specific primer/probe sets (Additional file 1: Figure S2A). We found that most MPNST cell lines had higher expression of JMa than JMb variants while CYT1 and CYT2 variant expression was comparable across our panel of cell lines (Additional file 1: Figure S2B and C).

### ErbB4 promotes proliferation, survival and angiogenesis in MPNSTs

**Fig. 2:**
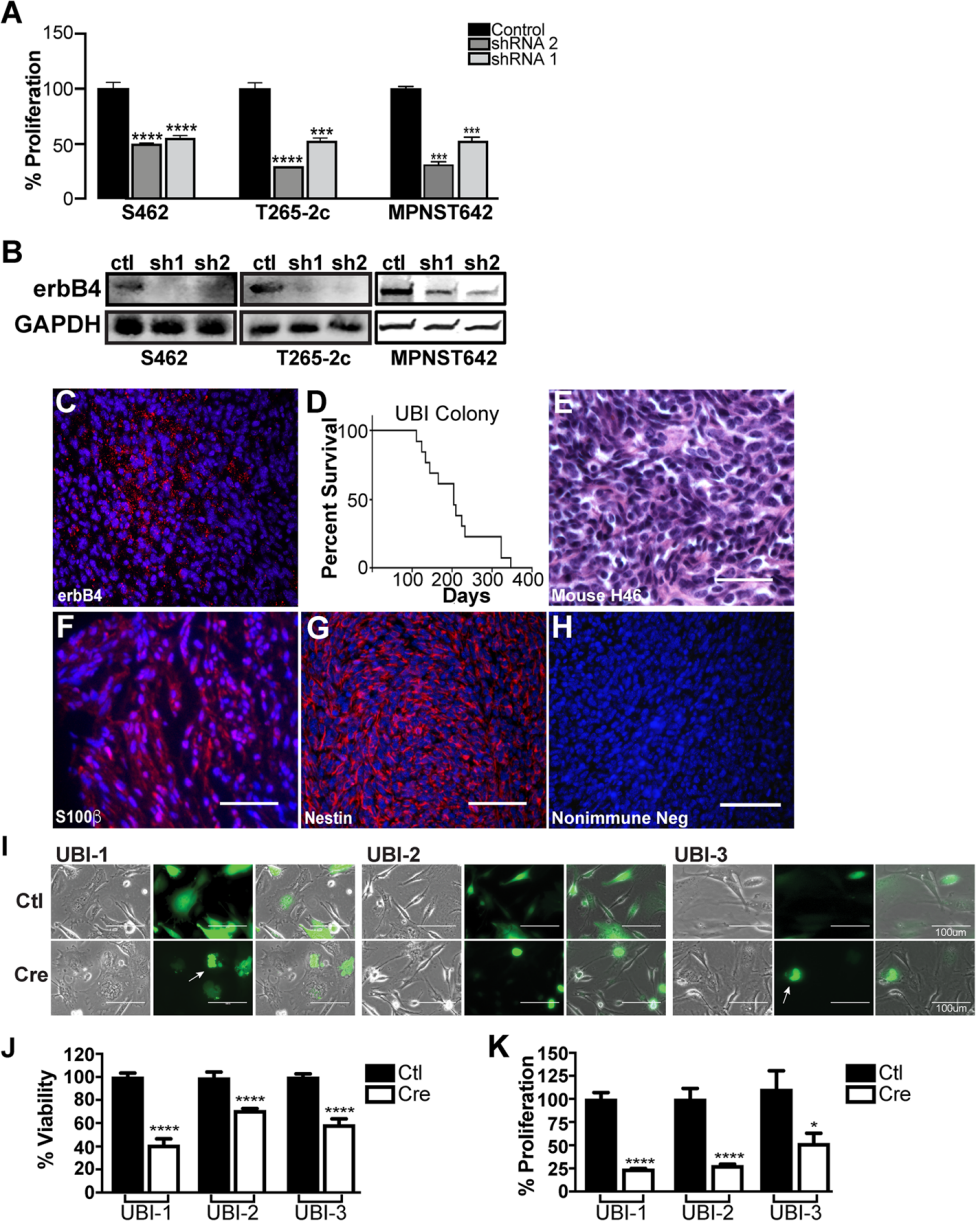
Loss of ErbB4 in human and mouse MPNST cells inhibits proliferation and survival. **a** Compared to non-targeting controls, erbB4 knockdown in cells had a decrease in cellular proliferation. **b** Immunoblots demonstrating reduced erbB4 expression in low erbB4 expressing cells (T265-2c, S462) and high expressing cells (MPNST642) cells infected with erbB4 shRNAs relative to cells infected with a non-targeting control. **, *p*-value≤0.01; ***, *p*-value≤0.001; ****, *p*-value≤0.0001. **c** Representative erbB4 immunostaining of P_0_-GGFβ3;*Trp53*^+/−^;*Erbb4*^*flox/flox*^ GEM mouse tumors showing erbB4 positivity compared to isotype matched negative controls (**h**). These representative erbB4 immunostains also show punctate membranous staining, with immunoreactivity present in almost all tumor cells (4+ staining). **d**) Kaplan-Meier survival curve of ^*/flox*^ mice shows that these animals survive an average of 210 days, similar to the parent line P_0_-GGFβ3;*Trp53*^+/−^ mice. **e**) Like their parent line, P_0_-GGFβ3;*Trp53*^+/−^;*Erbb4*^*flox/flox*^ mice develop hypercellular neoplasms comprised of atypical spindled cells with numerous mitotic figures. As in human MPNSTs, these tumors are also immunoreactive for both S100β (**f**) and nestin (**g**). **h** Isotype-matched negative control for erbB4 staining of mouse tumors. **i** Representative infection efficiency of UBI MPNST cell lines. MPNSTs with intact *Erbb4* alleles (control, non-recombined) have a spindled morphology similar to that of wild-type Schwann cells, whereas knockout cells (Cre) are more ameboid. Nuclear blebbing was evident in a subset of *Erbb4-*ablated tumor cells (arrow). Images were imaged at 40X on Brightfield and GFP channels. Infected cells are represented on the green channel due to GFP target sequence in the adenovirus. **j**, **k** Decreased cellular viability (**j**) and proliferation (**k**) was observed in the *Erbb4* knockout cells (Cre) compared to the control cells from three independent tumor cultures. *, *p*-value≤0.05; ****p-value≤0.0001 for comparisons of recombined (*Erbb4 -)* with non-recombined (*Erbb4+)* alleles. Red channel (Alexa 565, erbB4). Blue channel (Hoechst, nuclei)

To determine whether erbB4 promotes the proliferation and survival of human MPNST cells, we targeted *ERBB4* expression using shRNAs in cell lines (S462, T265-2c, and MPNST642) with increased erbB4 mRNA and protein expression compared to Schwann cells and assessed total cell number over a 5-day period of growth (Fig. 2a and b). Decreased erbB4 expression was confirmed by western blot; in these cells, we found that proliferation was also inhibited by loss of erbB4 expression. These findings were further supported by both MTT and ^3^H-thymidine incorporation assays in MPNST cells transiently transfected with pooled siRNA *ERBB4* or nonsense control siRNAs; *ERBB4* knockdown in these cultures showed smaller numbers of viable cells relative to cultures receiving nonsense control siRNAs (Additional file 1: Figure S3A-C), a change due, at least in part, to decreased proliferation.

Our previously described P_0_-GGFβ3;*Trp53*^+/−^ genetically engineered mouse (GEM) model develops MPNSTs de novo with complete penetrance [20]; like human MPNSTs, these GEM tumors and early passage cultures derived from them express erbB4 (Fig. 2c; Additional file 1: Figure S2D). We therefore decided to use this model to further dissect erbB4’s role in MPNST pathogenesis in vivo. Unfortunately, however, we could not generate P_0_-GGFβ3;*Trp53*^*+/−*^ mice with germline *Erbb4* deletion as germline *Erbb4* loss produces cardiac defects that result in embryonic lethality [33]. The approach of introducing floxed *Erbb4* alleles into MPNSTs and deleting them with CreER^T2^ was also problematic as our initial experiments indicated that mosaicism would complicate the interpretation of these experiments. We therefore tried crossing P_0_-GGFβ3;*Trp53*^*+/−*^ mice to mice in which *Erbb4*^*−/−*^ lethality is rescued by re-expressing erbB4 in the heart (*Erbb4*^−/−^;α-MHC-*Erbb4* mice). However, out of 65 pups expected to have the desired P_0_-GGFβ3;*Trp53*^*+/−*^*;Erbb4*^−/−^;α-MHC-*Erbb4* genotype, we obtained only one pup with this genotype and it did not produce any progeny.

We thus took the alternative approach of producing P_0_-GGFβ3;*Trp53*^*+/−*^*;Erbb4*^*flox/flo*x^ mice, with the goal of deleting *Erbb4* ex vivo, grafting the cells back into mice and then assessing the effects of *Erbb4* ablation on tumor growth. The survival of P_0_-GGFβ3;*Trp53*^*+/−*^*;Erbb4*^*flox/flo*x^ mice (average survival, 210 days; Fig. 2d) was indistinguishable from that of P_0_-GGFβ3;*Trp53*^*+/−*^ mice [20]. Peripheral nervous system tumors were present in nearly 100% of the P_0_-GGFβ3;*Trp53*^*+/−*^*;Erbb4*^*flox/flo*x^ mice; these tumors were most commonly associated with trigeminal nerves, with some evident in sciatic nerve or dorsal root ganglia (Additional file 1: Table S2). The tumors in P_0_-GGFβ3;*Trp53*^*+/−*^*;Erbb4*^*flox/flo*x^ mice were markedly hypercellular MPNSTs with brisk mitotic activity (Fig. 2e) and immunoreactivity for S100β and nestin (Fig. 2f-h). As tumor penetrance and lifespan in P_0_-GGFβ3;*Trp53*^*+/−*^;*Erbb4*^*flox/flox*^ mice and P_0_-GGFβ3; *Trp53*^*+/−*^; mice was similar, we concluded that the floxed *Erbb4* alleles did not impede tumorigenesis or worsen the survival of these mice.

To determine what effects erbB4 loss had in these tumors, we established early passage P_0_-GGFβ3;*Trp53*^*+/−*^;*Erbb4*^*flox/flox*^ MPNST cultures and transduced them with adenovirus expressing eGFP or eGFP-Cre recombinase. eGFP-positive cells were isolated via FACS from each treatment group. PCR analyses verified the presence of recombined *Erbb4* alleles and *Erbb4* mRNA reductions (Additional file 1: Figure S3D and E) in the presence of Cre recombinase, but not in controls. *Erbb4* ablation did not reduce the expression of *Egfr*, *Erbb2* or *Erbb3* mRNAs (Additional file 1: Figure S3F) indicating that the changes described below are unlikely to reflect indirect effects on the expression of other erbB family members. The purified tumor cells were viable but demonstrated morphologic changes. In contrast to the spindled morphology of cells with intact *Erbb4* alleles (Fig. 2i), the *Erbb4* knockout cells were ameboid with shorter, thicker processes and often showed nuclear blebbing (Fig. 2i. GFP vs Cre). To determine whether *Erbb4* ablation in P_0_-GGFβ3;*Trp53*^*+/−*^;*Erbb4*^*flox/flox*^ MPNSTs inhibited viability, we performed MTT assays on transduced cultures of three independently arising MPNSTs. We found that cultures transduced with Cre-expressing adenovirus showed decreased numbers of viable cells compared to cells infected with the control vector (Fig. 2j). As in human MPNST cells, ^3^H-thymidine incorporation assays demonstrated that this decrease in cell numbers was due, in part, to reduced proliferation (Fig. 2k).

**Fig. 3:**
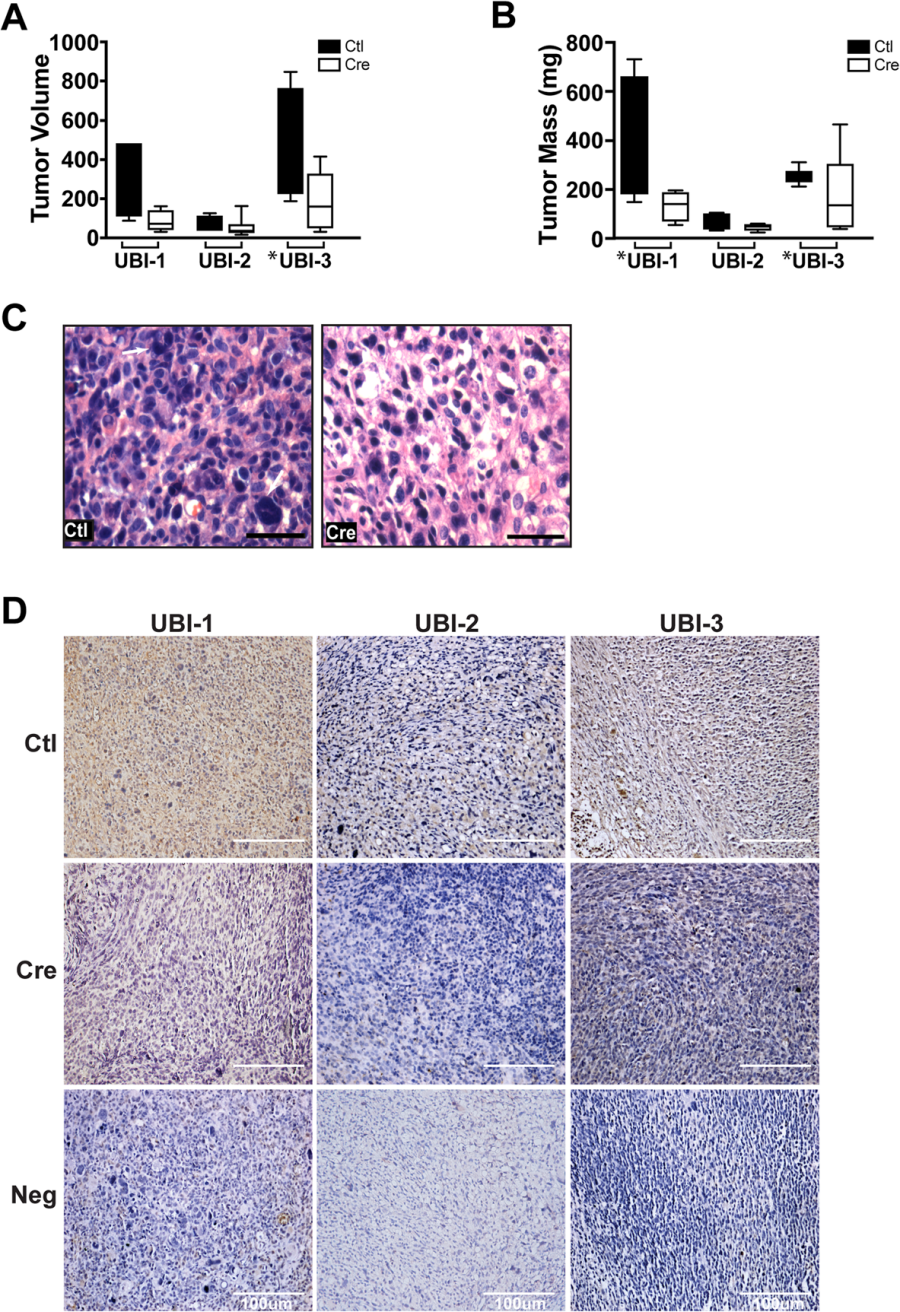
*Erbb4* ablation inhibits allograft growth and alters tumor histopathology in Nude mice. **a** and **b**) *Erbb4-*null cells from three separate tumor lines show reduced graft volume (**a**) and mass (**b**) compared to erbB4 expressing controls. *p*-values ≤0.05 are designated with an asterisk (*), Tumor mass *p*-values are as follows: UBI-1: 0.049, UBI-2: 0.069, and UBI-3: 0.005; F-Test. **c** Representative histologic images of a UBI GFP control tumor and a UBI Cre tumor taken at 40X. Arrows in Ctl indicate the multinucleated cells that are commonly seen in these tumors. Note that these multinucleated cells are virtually absent in the *Erbb4*-null grafts. **d** Representative chromogenic erbB4 stained images of UBI GFP control tumor and UBI Cre tumors displaying decreased erbB4 expression in Cre ablated xenografts compared to control tumors taken at 20X

To examine the effects of *Erbb4* ablation in vivo, the three distinct P_0_-GGFβ3;*Trp53*^*+/−*^*;Erbb4*^*flox/flo*x^ early passage MPNST cultures previously assessed by MTT and ^3^H-thymidine incorporation assays (Fig. 2j-k) were transduced with eGFP- or eGFP-Cre expressing adenovirus, FACs sorted and then orthotopically allografted in the sciatic nerves of nude mice. The grafts were excised 35 days post-grafting, and their masses and volumes determined. Grafts of *Erbb4*-ablated tumor cells showed overall reduction in volume and mass relative to grafts of cells transduced with eGFP-expressing adenovirus (Fig. 3a and b). The cellularity of the *Erbb4*-null tumors was lower than that of the eGFP controls (Fig. 3c, Ctl vs Cre) and the *Erbb4* knockout cells had more prominently vacuolated cytoplasm. The expression of erbB4 in the resultant tumors was assessed in several excised tumors from each UBI cohort. Expression of erbB4 was found to be lower in Cre erbB4 ablated tumors versus Ctl erbB4 expressing tumors (Fig. 3d, Ctl vs Cre). The degree of erbB4 expression was slightly variable between data sets but is supportive in the trend observed with tumor mass/volume data. Our immunohistochemical data thus supports the hypothesis that the larger tumors we observed in our Cre-mediated knockout cohorts likely resulted from the presence of erbB4 expressing cells that escaped Cre-mediated gene ablation rather than a lack of dependence on erbB4.

**Fig. 4:**
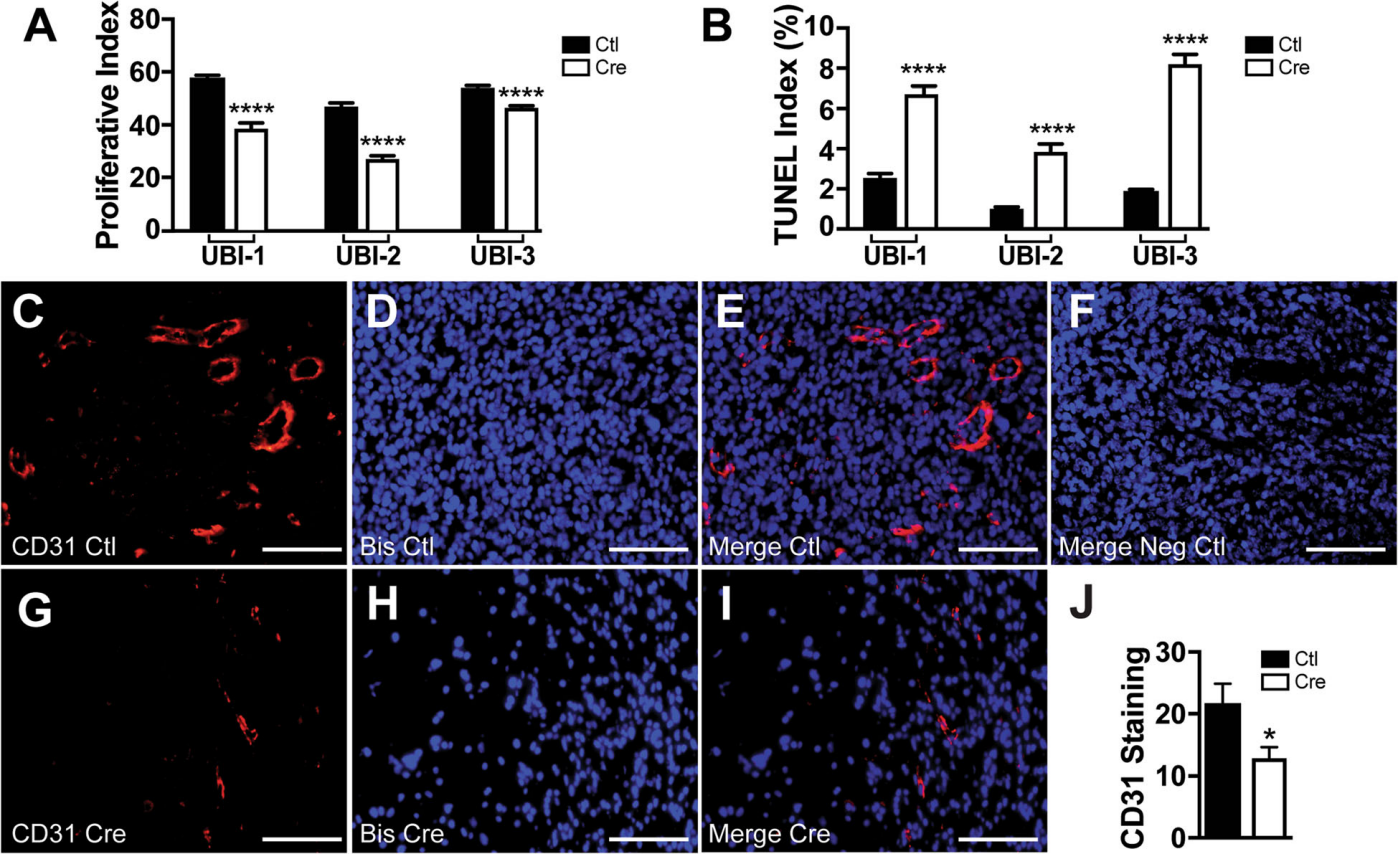
**a** Quantification of Ki67 labeling indices in three allografted tumor lines. **b** Quantification of TUNEL labeling indices in the same tumor lines. ****, *p*-value≤0.0001 for comparisons of recombined *Erbb4* allele (erbB4 -) grafts to grafts with non-recombined *Erbb4* alleles (erbB4+). **c**-**i** Representative CD31 staining of control erbB4 positive tumors (**c**, **e**) and erbB4 negative tumors (**g**, **i**) with Bisbenzimide counterstain (**b**, **h**) for cell nuclei detection. A non-immune isotype matched primary was used as a negative control (**f**). Images were acquired at 40X. Scale bars represent 100 um. **j** Quantification of representative immunofluorescent images using FIJI. *, *p*-value≤0.05; ****p-value≤0.0001 for comparisons of recombined (*Erbb4 -)* with non-recombined (*Erbb4+)* alleles

To determine whether this decrease in graft mass and volume reflected decreased tumor cell proliferation or survival, we quantified the Ki67 and TUNEL labeling indices in five *Erbb4*-ablated and five control grafts established from each of the three early-passage cultures. In all instances, there were statistically significant decreases in Ki67 labeling in the *Erbb4*-null grafts relative to eGFP controls (Fig. 4a). In addition, the *Erbb4*-ablated allografts demonstrated a 3–4-fold increase in the percentage of TUNEL positive cells relative to the control grafts (Fig. 4b). *Erbb4* loss thus results in both decreased proliferation and increased apoptosis in these grafts.

During our gross examination of the grafts, we noted that the *Erbb4* ablated grafts were paler than the controls, raising the question of whether vascular density was decreased in the grafts lacking *Erbb4*. Upon immunostaining our grafts for the vascular marker CD31, we found that the vascular density in the *Erbb4* ablated grafts (Fig. 4g-i) was lower than that in the control allografts [(Fig. 4c-e; a nonimmune control primary antibody was used as a negative control (Fig. 4f)]. Quantification of the vascular densities in these grafts showed that the vascular density in the *Erbb4* ablated grafts was approximately half that of the control grafts (Fig. 4j). As *Erbb4* is intact in the vasculature within these grafts, this suggests that the decreases in vascular density observed in *Erbb4* ablated grafts are an indirect effect of *Erbb4* loss in MPNST cells via mechanisms such as erbB4-mediated enhancement of the production and/or secretion of angiogenic factors by MPNST cells.

### ErbB4 mediates basal and NRG1-stimulated phosphorylation of multiple cytoplasmic kinases, but is not essential for Ras hyperactivation

**Fig. 5:**
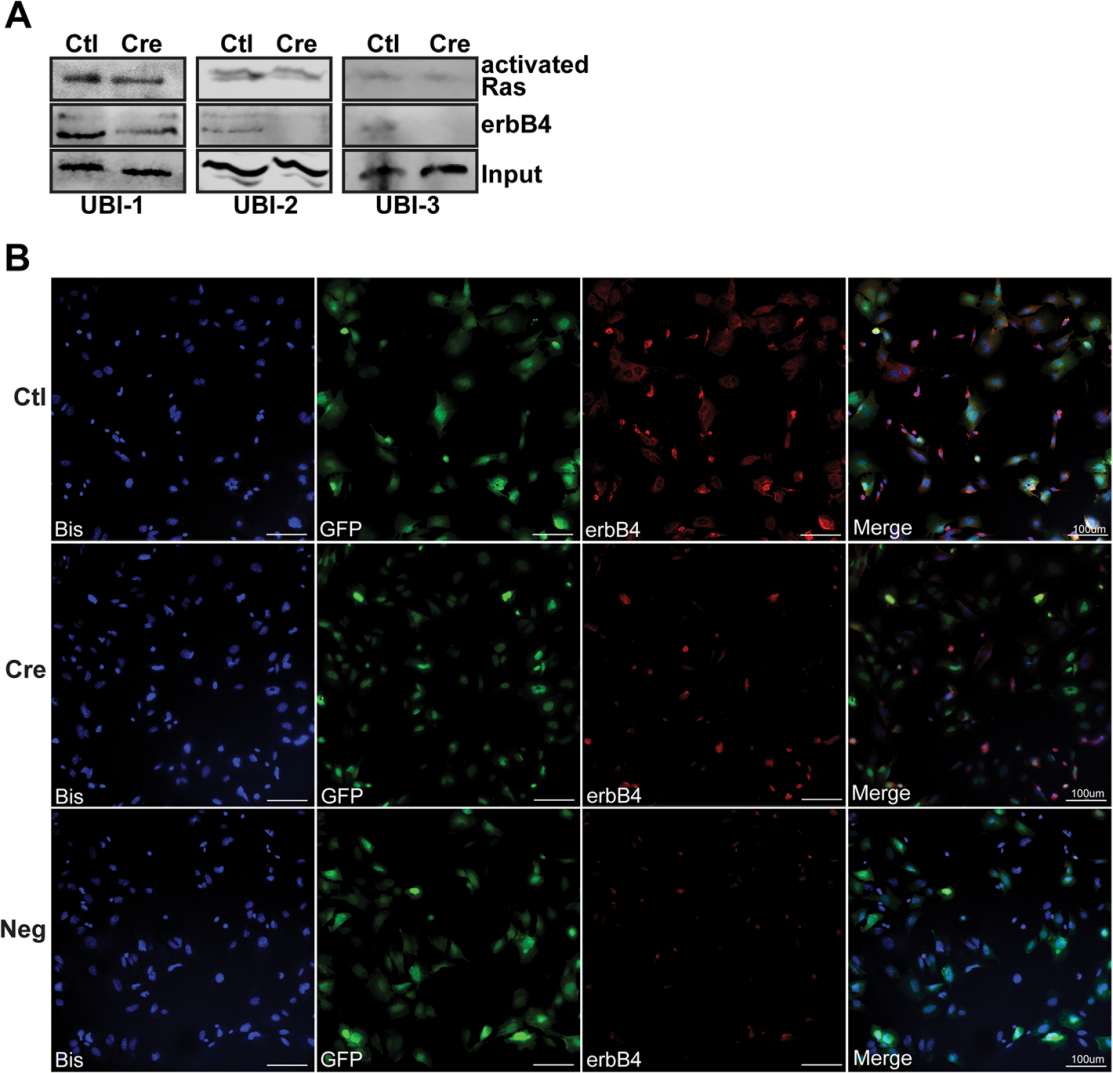
*Erbb4* is not essential for Ras hyperactivation. **a** A comparison of Ras activation in *Erbb4*-null (Cre) and *Erbb4*-expressing control (Ctl) UBI 1–3 MPNST cells shows that *Erbb4* loss does not affect Ras activation. **b** Immunocytochemistry staining of *Erbb4*-null (Cre) and *Erbb4*-expressing control (Ctl) UBI MPNSTs cells displays reduced erbB4 expression in adeno-viral infected cells (GFP positive). Representative erbB4 staining of control erbB4 positive tumors (Ctl) and erbB4 negative tumors (Cre) with Bisbenzimide counterstain for cell nuclei detection. A non-immune isotype matched primary was used as a negative control. Images were acquired at 20X. Scale bars represent 100 um

Although MPNSTs co-express erbB3 and erbB4, our demonstration that *Erbb4* ablation decreased proliferation, survival and angiogenesis in MPNSTs indicates that these receptors are not functionally redundant and suggest that erbB4 activates distinct signaling cascades essential for MPNST tumorigenesis. As many growth factors, including NRG1 [34], activate Ras, we first asked whether *Erbb4* ablation diminished the erbB-dependent Ras hyperactivation we have previously observed in P_0_-GGFβ3;*Trp53*^*+/−*^ MPNSTs [20]. To answer this question, we generated lysates from early passage P_0_-GGFβ3;*Trp53*^*+/−*^*;Erbb4*^*flox/flo*x^ MPNST cells transduced with eGFP or eGFP-Cre expressing adenovirus, pulled down the activated Ras proteins with Raf-1 and then probed the captured Ras proteins using a pan-Ras antibody (Fig. 5a). Surprisingly, we found that Ras activation was not reduced by *Erbb4* ablation, indicating that *Erbb4* loss impairs MPNST proliferation and survival via effects on Ras-independent pathways. Immunocytochemistry confirmed reduced erbB4 expression in adeno-Cre-GFP infected cells compared to adeno-Cre-GFP infected cells (Fig. 5b), indicating that continued Ras activation did not reflect a failure of erbB4 ablation.

**Fig. 6:**
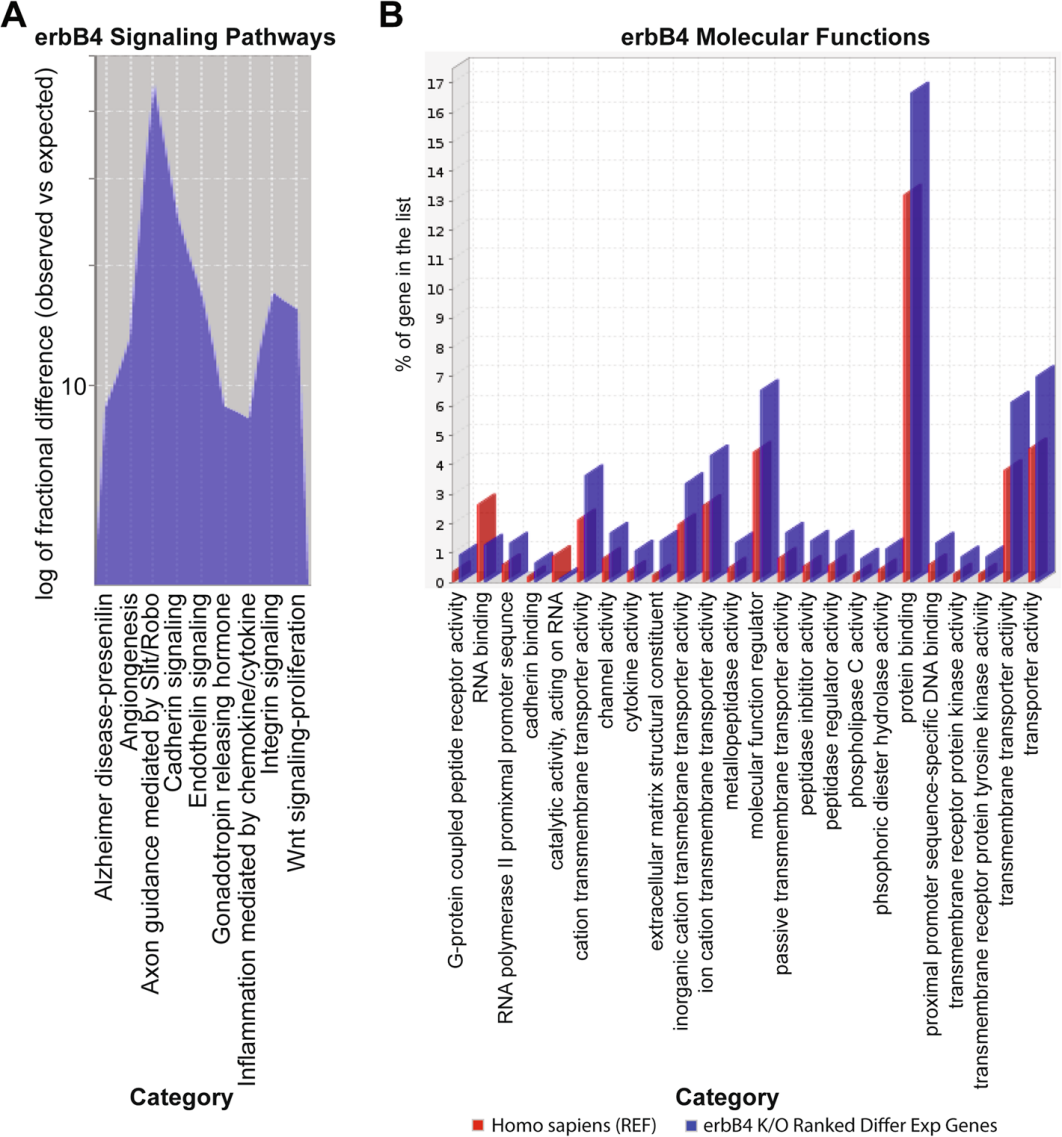
RNA-Seq pathway analysis of differentially affected genes in erbB4 knockout UBI MPNST cells. Partek analysis of NextGen RNA sequencing identified differentially expressed genes. Genes with a fold change of at least 1.5 up or down were put through Panther gene over representation enrichment analysis to identify erbB4 affected signaling pathways (**a**) and molecular function activity (**b**). Graphs represent the ratio of over-represented genes differentially affected by erbB4 loss compared to expected reference representation. This analysis highlights the role of erbB4 in migration, angiogenesis and PLCγ signaling

As a further assessment of the biological functions impacted by *Erbb4* loss, we performed RNA-Seq on UBI-1, 2, and 3 cell lines transduced with GFP- or Cre/GFP-expressing adenovirus, identified the genes whose expression was significantly changed ≥1.5-fold and performed gene ontology analyses on these genes. Using Panther over-represented analysis tools [35] to identify the top pathways (Fig. 6a) and molecular function activities (Fig. 6b) affected by *Erbb4* loss, we identified angiogenesis, adhesion, proliferation and cation signaling as key erbB4-regulated functions. These findings were thus consistent with the in vitro and in vivo observations described above.

To further explore the erbB4 mediated signaling driving proliferation, survival and angiogenesis in MPNSTs, we stimulated *Erbb4* intact and *Erbb4*-null GGFβ3;*Trp53*^*+/−*^*;Erbb4*^*flox/flo*x^ MPNST cells with 10 nM NRG1β for 5 min, a time that our preliminary analyses indicated was sufficient to maximally activate NRG1β-responsive signaling pathways. Lysates from these cells were used to probe arrays containing phosphorylation site-specific antibodies that query 43 different cytoplasmic kinases and other key proteins (Additional file 1: Figure S4). We then asked which of these proteins showed altered phosphorylation following NRG1β stimulation and *Erbb4* ablation.

**Fig. 7:**
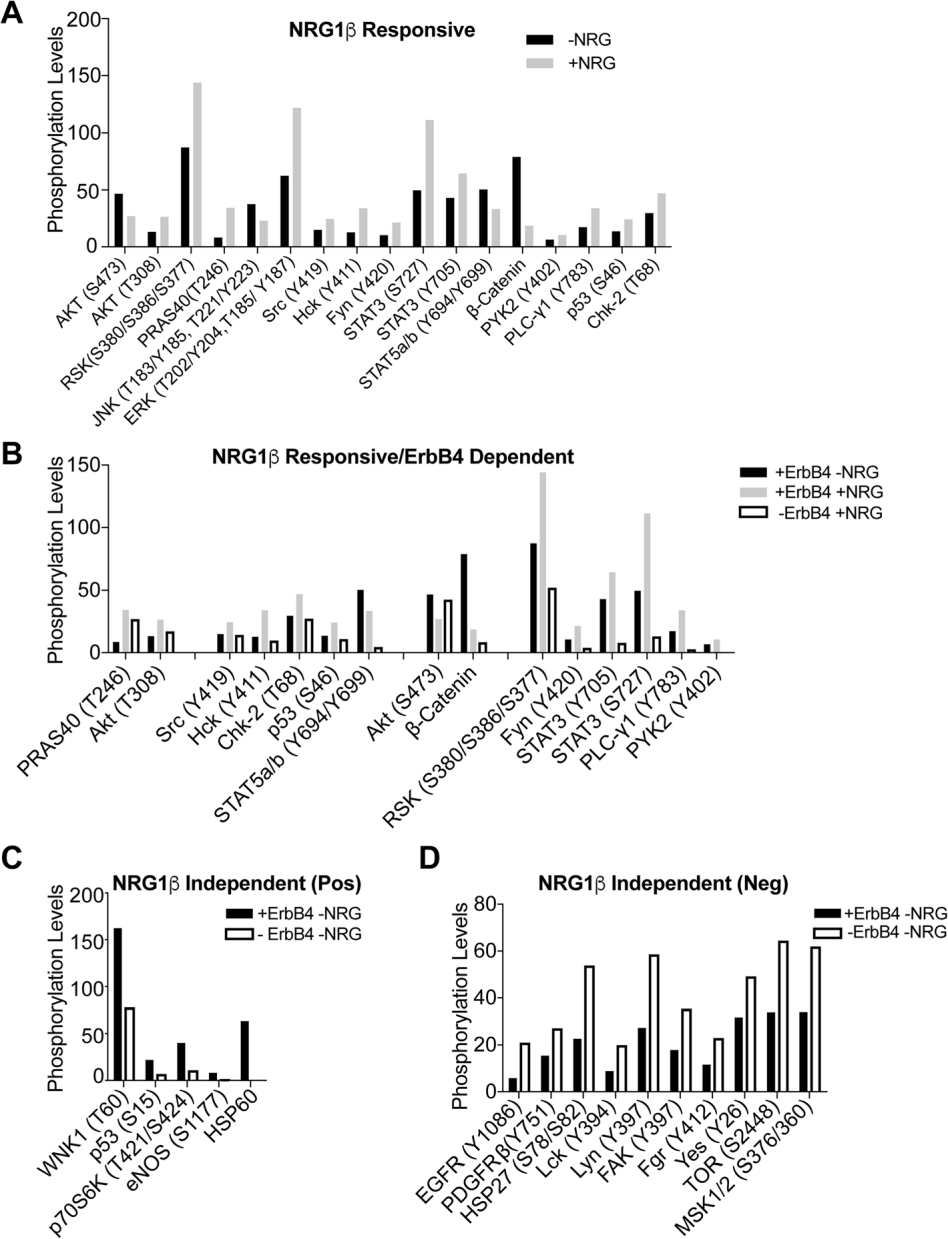
*Erbb4* increases the phosphorylation of a number of other cytoplasmic kinases, independent of Ras activation. **a** Graphical representation of quantified kinase arrays comparing the levels of baseline phosphorylation to NRG1 stimulated phosphorylation in *Erbb4*-expressing UBI MPNST cells. The graph includes the subset of kinases whose phosphorylation was altered following NRG1β stimulation. **b** Graphical representation of quantified kinase arrays comparing the levels of baseline phosphorylation of control *Erbb4-* intact cells to NRG1 stimulated *ErbB4*-intact and *Erbb4-null* UBI MPNST cells to determine NRG1 dependent and *ErbB4* dependent kinases. **c**) Quantification of a subset of the non-responsive kinases *ErbB4*-intact compared to *ErbB4*-ablated to identify targets positively regulated by *ErbB4* independent of stimulation. **d**) Quantification of a subset of the non-responsive kinases *ErbB4*-intact compared to *ErbB4*-ablated to identify compensatory targets resulting from *ErbB4*-ablation independent of stimulation. Quantification of the kinases differentially phosphorylated was quantified per the manufacturer’s protocol using ImageJ

We first identified the NRG1β responsive proteins and kinases in *Erbb4* intact GGFβ3;*Trp53*^*+/−*^*;Erbb4*^*flox/flo*x^ MPNST cells; as NRG1β activates both erbB3 and erbB4, alterations in the phosphorylation of the proteins queried by our arrays in these cells could reflect the action of either erbB3 or erbB4. Kinases whose phosphorylation levels were changed by at least 1.5-fold compared to unstimulated cells were considered NRG1β responsive. Using this criterion, we identified 13 NRG1β responsive sites with increased phosphorylation and 4 NRG1β responsive events in which the phosphorylation of the targeted protein was decreased (Fig. 7a). The proteins in which NRG1β increased phosphorylation included Akt (T308), RSK, PRAS40 [also known as AKT1S1 (Akt1 substrate 1)], Src, Hck, Fyn, STAT3 (S727 and Y705), PYK2, PLCγ1, p53 (S46) and Chk-2; of particular note, given that we have previously shown that Ras is activated in P_0_-GGFβ3;*Trp53*^+/−^ MPNSTs by NRG1 [20], we found that the phosphorylation of ERK was also increased by NRG1β stimulation in these experiments. In contrast, the phosphorylation of S473 in pAKT (unlike T308, another activation site in Akt, where phosphorylation was increased by NRG1β; see above), JNK, STAT5a/b (Y694/Y699) and β-catenin was decreased following NRG1β stimulation. However, since decreased phosphorylation of β-catenin results in increased transcriptional activity, this latter change reflects increased activation of the β-catenin pathway downstream of NRG1β.

To determine which phosphorylation events noted above required NRG1β-mediated activation of erbB4, we compared the stimulated phosphorylation profile of *Erbb4* intact cells to that of *Erbb4* ablated cells (Fig. 7b). We found that two of the 18 NRG1β-responsive phosphorylation events were partially dependent on the presence of *Erbb4* (PRAS40, Akt-T308) suggesting that erbB3 and erbB4 redundantly regulate these PI3K pathway proteins downstream of NRG1β through distinct heterodimers. The phosphorylation of six kinases (Src, Hck, Chk-2, p53-S46, STAT5a and b) was completely dependent on NRG1β and intact *Erbb4*, indicating that NRG1β-driven phosphorylation of these targets is mediated, directly or indirectly, by erbB4. Interestingly, several of the changes we observed suggested that erbB3 and erbB4 play opposing roles in activating some signaling pathways in MPNSTs. For instance, the phosphorylation of STAT5a/b at Y694/699, two sites that are required for activation, was weakly (0.66 fold down) decreased by NRG1β in the presence of *Erbb4*. However, in the absence of *Erbb4*, NRG1β stimulation resulted in an almost complete loss of STAT5a/b activation, suggesting that erbB4 opposes erbB3-mediated inhibition of STAT5a/b. The reduction in pAkt-S473 phosphorylation observed in response to NRG1β stimulation (Fig. 7a) was also rescued by *Erbb4* ablation, with pAkt-S473 returning to baseline levels in the presence of NRG1β (Fig. 7b). In contrast, the reduction in β-catenin phosphorylation levels observed in response to NRG1β stimulation was further exaggerated when *Erbb4* was ablated, suggesting both erbB3 and erbB4 activate this signaling cascade. The remaining six NRG1β -responsive proteins (RSK, Fyn, STAT3-Y705/S727, PLCγ-1, PYK2) had phosphorylation that was enhanced by erbB4, both in the presence and absence of exogenous NRG1β (Fig. 7b). This latter observation may reflect erbB4 activation mediated by endogenous NRG1β expressed by the tumor cells themselves or, alternatively, the action of other erbB4 ligands expressed by the MPNST cells that similarly activate downstream signaling cascades.

We next asked whether there were additional proteins whose phosphorylation was dependent upon *Erbb4* but unaffected by NRG1β stimulation. We identified 15 proteins whose phosphorylation was not altered when challenged with exogenous NRG1β but that were sensitive to *Erbb4* ablation. *Erbb4* ablation decreased the phosphorylation of five of these proteins [WNK1, p53 (S15), p70S6K, eNOS, and HSP60; Fig. 7c), suggesting that *Erbb4* is a key upstream activator of these kinases. *Erbb4* loss increased the phosphorylation of the other ten proteins in a NRG1β independent manner suggesting that erbB4 negatively regulates the activation of these proteins. This latter group of proteins included two RTKs, EGFR and PDGFRβ, as well as HSP27, five Src-related kinases (Lck, Lyn, FAK, Fgr, Yes), mTOR and MSK1/2 kinases (Fig. 7d). The majority of these proteins are associated with the plasma membrane and their activation in the absence of erbB4 may be a direct result of altered receptor heterodimerization and cellular architecture at the plasma membrane.

### MPNSTs are sensitive to inhibitors targeting erbB4 activated signaling pathways

**Fig. 8:**
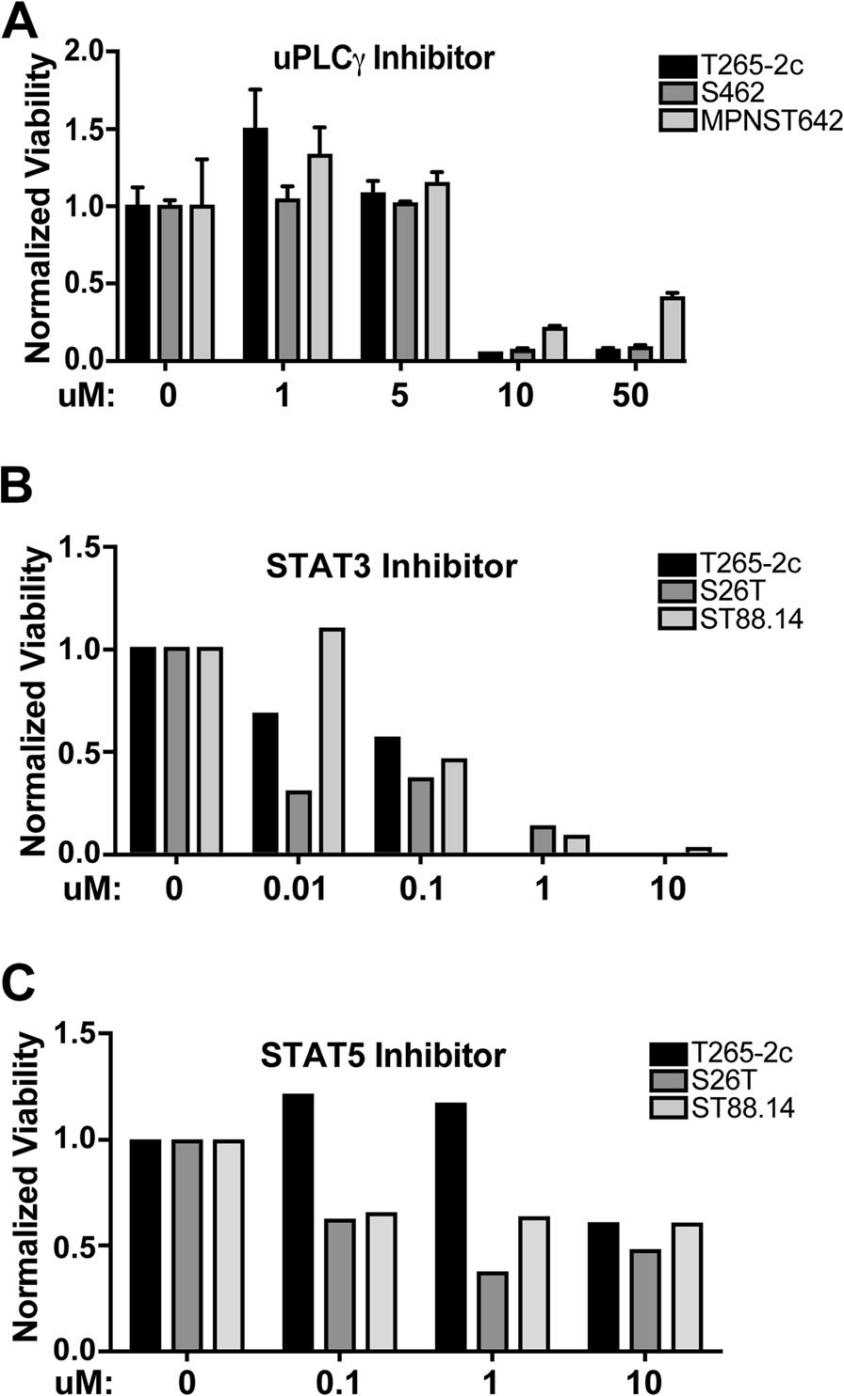
MPNSTs are sensitive to inhibition of specific erbB4 activated signaling pathways. **a**-**c** Cell viability was assessed in three log phase MPNST cell lines in the presence of designated inhibitor. Values represent cell viability after 72 h of drug treatment normalized to 0 h. After normalization, cell viability for each drug dose was compared to vehicle control. PLC-γ inhibition effects cell viability in a dose dependent manner **a**. STAT3 inhibition significantly affects cell viability (**b**). STAT5 inhibition modestly affects cell viability (**c**)

**Fig. 9:**
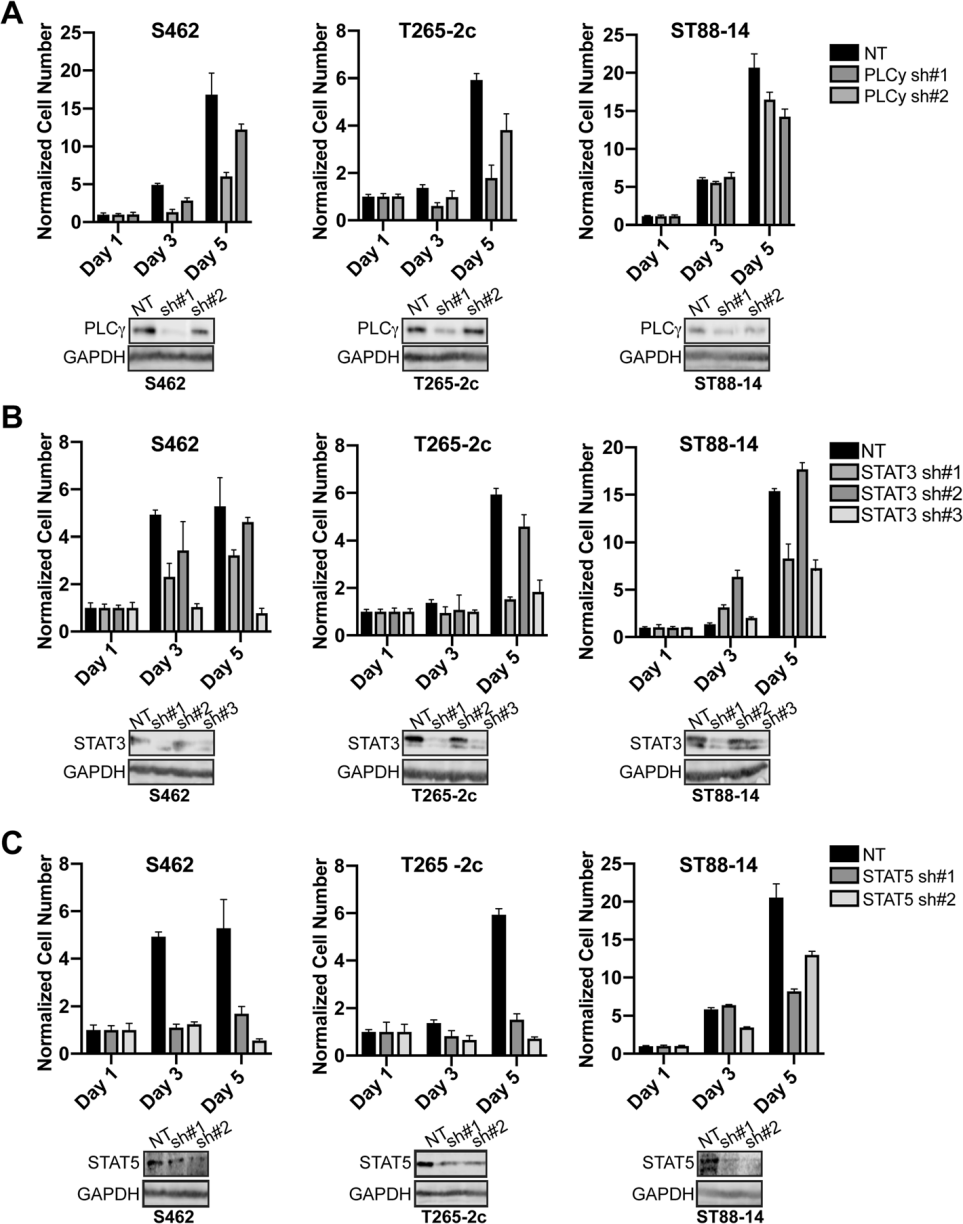
MPNSTs are sensitive to knockdown of specific erbB4 activated signaling molecules. **a**-**c**) Cell proliferation was assessed in three log phase MPNST cell lines in the presence of multiple designated PLCγ(**a**), STAT3 (**b**), or STAT5 (**c**) shRNA’s or non-targeting controls. Values represent cell number over 5 days of shRNA knockdown normalized to 0 h. PLC-γ inhibition effects cell proliferation in a cell-type dependent manner (**a**). STAT3 inhibition significantly affects cell proliferation (**b**). STAT5 inhibition significantly affects cell proliferation (**c**)

**Fig. 10:**
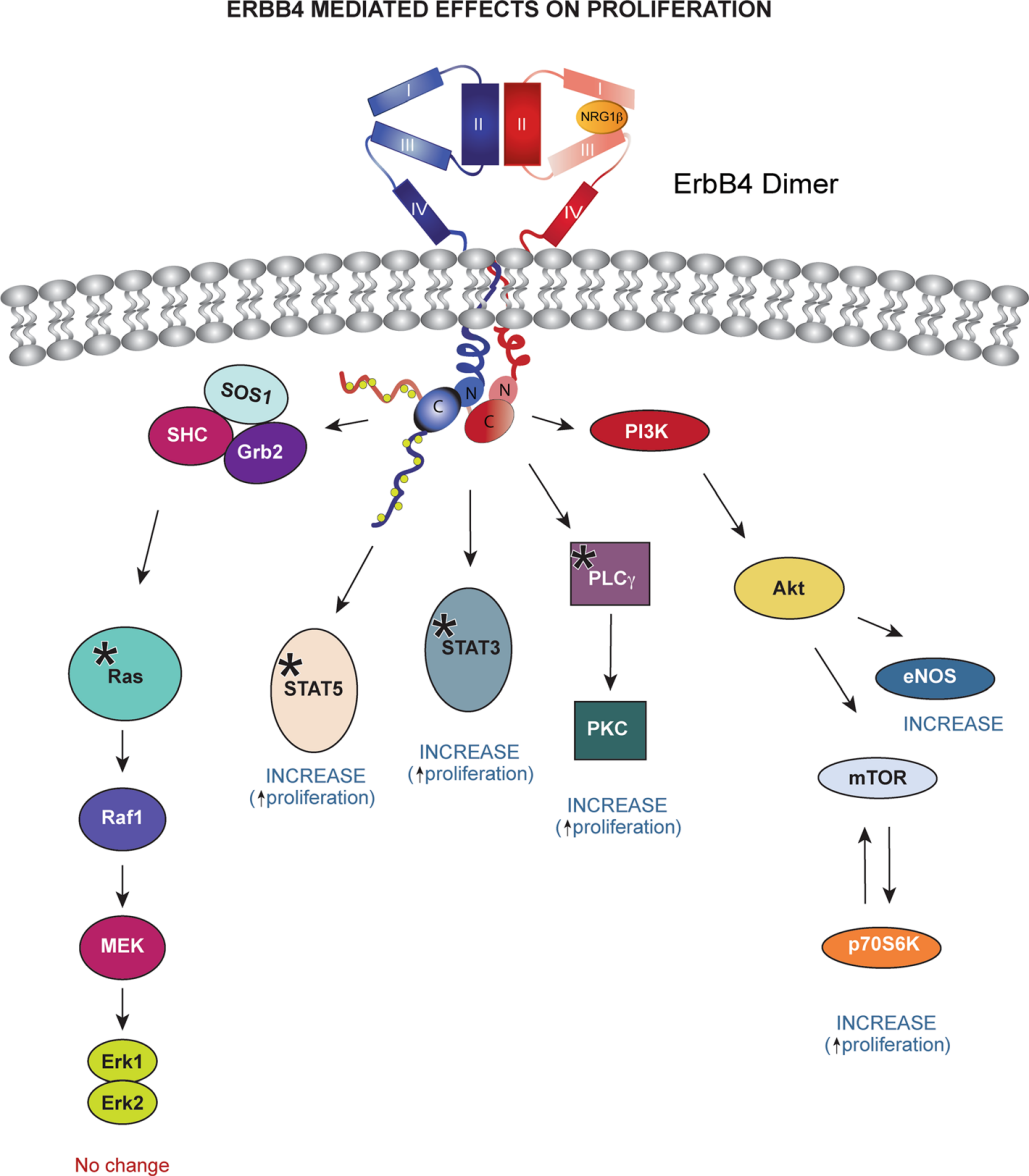
Schematic highlighting the signaling cascades that are dependent on erbB4 in MPNSTs. The STAT3/5 and PI3K pathway is positively regulated by erbB4 in a NRG1β-dependent manner. Kinases validated experimentally are demarcated with an asterisk

To directly assess whether the proteins whose phosphorylation is altered by *Erbb4* loss include molecules that promote MPNST proliferation and/or survival, we inhibited the actions of four of these proteins (WNK1, PLCγ, STAT3 and STAT5a). Although our shRNAs effectively knocked down WNK1 expression in T265-2c and S462 cells, MTT assays did not show a reproducible decrease in the number of viable cells following knockdown (Additional file 1: Figure S5A). In contrast, the PLCγ inhibitor U73122 inhibited the viability of T265-2c, S462 and MPNST642 cells (Fig. 8a). Likewise, the STAT3 inhibitor Cas285986–31-4 also inhibited the viability of three MPNST cell lines in a concentration-dependent manner (Fig. 8b). The STAT5 inhibitor 5,15-DPP also decreased the number of viable cells in cultures of these lines, although its effects were not as pronounced as those of the STAT3 inhibitor (Fig. 8c). To further confirm these observations, at least two to three gene specific shRNAs against PLCγ, STAT3 and STAT5 were employed in a proliferation assay over a five-day growth period to challenge these drug studies. Knockdown of PLCγ, STAT3, and STAT5 resulted in a decrease in total cell number in a cell-type dependent manner (Fig. 9a). S462 and T265-2c were sensitive to decreased PLCγ expression and displayed a decrease in total cell number. ST88–14 cells had a less pronounced effect on cell number, likely due to poor PLCγ knockdown efficiency. STAT3 and STAT5 shRNA mediated knockdown both downregulated MPNST total cell number in all three cell lines in protein expression dependent manner (Fig. 9b and c). We conclude that PLCγ, STAT3 and STAT5 are among the key erbB4 regulated targets to mediate the proliferation and/or survival of MPNST cells (Fig. 10).

## Discussion

The erbB4 receptor tyrosine kinase has been implicated in the pathogenesis of several human cancers including glioblastomas [36], melanomas [37, 38], medulloblastomas [39], pulmonary adenocarcinomas [40], ovarian carcinomas [41] and esophageal carcinomas [42]. Many of these cancer types also express erbB3, a second NRG1 receptor that is functionally distinct from erbB4. Surprisingly, it has not been clearly established whether the co-expression of two NRG1 receptors in these neoplasms promotes tumorigenesis and, if they do, whether they do so by increasing receptor numbers (thereby rendering tumor cells more responsive to NRG1 or other erbB ligands) or whether these two receptors instead make distinct, critically important contributions to tumor initiation and progression. The evidence we have obtained using MPNSTs as a model system argues that, at least in this setting, erbB3 and erbB4 promote tumorigenesis by regulating different essential signaling cascades. However, our findings also raise intriguing new questions regarding the mechanisms by which erbB4 drives tumor pathogenesis.

Several lines of evidence reported in this manuscript support the hypothesis that erbB4 promotes MPNST pathogenesis. Our immunohistochemical, real time PCR and immunoblotting results demonstrate that multiple erbB4 splice variants, including variants capable of activating PI3K signaling and mediating transcription, are expressed in the overwhelming majority of human and mouse MPNSTs. Ablation of *Erbb4* in P_0_-GGFβ3;*Trp53*^*+/−*^;*ErbB4*^*flox/flox*^ MPNST cells results in altered cellular morphology, increased cell death and reduced proliferation. Further, when allografted in nude mice, *Erbb4*-null P_0_-GGFβ3;*Trp53*^*+/−*^;*Erbb4*^*flox/flox*^ MPNST allografts produce tumors up to 80% smaller than controls with evidence of decreased mitogenesis, increased cell death and decreased vascular density. Finally, knocking down *ERBB4* expression in human MPNST cells has effects on proliferation and survival analogous to those seen in *Erbb4*-null mouse MPNSTs. We found that loss of erbB4 expression did not reduce the expression of erbB3, the other NRG1 receptor, in these MPNST cells. Further, the *Erbb4*-null tumor cells are still NRG1-responsive, as demonstrated by our phosphorylation-specific antibody array experiments. It is thus apparent that the erbB3 receptors expressed in MPNST cells cannot completely compensate for the loss of erbB4 expression.

Our findings also indicate that erbB3 and erbB4 mediate distinct effects in MPNST cells. The erbB3 receptors expressed in MPNST cells are clearly functional as the phosphorylation of the Ras downstream effectors Erk-1/2 and JNK-1/2/3 were similarly increased in NRG1β-stimulated *Erbb4*-null cells and unmodified controls. Consequently, the effects we observed following *Erbb4* ablation are not due to a loss of NRG1 responsiveness. These changes are also not the result of decreased Ras activation as Ras activation was unaffected by *Erbb4* loss. As both erbB3 and erbB4 can activate Ras, this suggests that erbB3 and/or other erbB receptors compensate for erbB4 loss in MPNST cells to maintain Ras activation. Our demonstration that erbB4 loss results in decreased proliferation and survival thus indicates that erbB4 must promote the proliferation and survival of MPNST cells by regulating other non-Ras dependent signaling cascades.

By comparing kinase phosphorylation in NRG-1β stimulated and unstimulated *Erbb4*-null and control cells, we identified several candidate kinases that potentially represent the downstream effectors of erbB4-mediated proliferation, survival, angiogenesis and, potentially, other effects in MPNSTs. In keeping with our demonstration that MPNST cells express erbB4 splice variants containing the Cyt1 domain, *Erbb4*-null MPNST cells demonstrated altered phosphorylation of Akt and key Akt targets such as, eNOS, ribosomal p70S6 kinase (S6K) and ribosomal p90S6 kinase (RSK1/2/3); the phosphorylation of these molecules was decreased in Cre-treated *Erbb4* ablated tumor cells, indicating that erbB4 positively regulates the PI3K/Akt/mTOR signaling cascades in MPNST cells. However, erbB-mediated regulation of Akt action in MPNST cells is likely complex. We have found that NRG1β, acting through erbB4, reduced the phosphorylation of Akt residue S473 yet we observed an increase in T308 phosphorylation that was at least partially dependent on erbB4. A similar inverse response on Akt has been previously reported in response to ER-stress and is a context dependent mechanism that diversifies Akt signaling [43]. Although the phosphorylation of both of these sites activates Akt, the phosphorylation of these two amino acids is orchestrated by different kinases; S473, which resides in the regulatory domain of Akt, is phosphorylated by mTORC2 and DNA-PK, while T308 which is located in the Akt kinase domain, is a direct target of PDK1. The fact that both S473 and T308 were found to be increased by NRG1 stimulation in the absence of erbB4 suggest that this increase is mediated partially by erbB3. The fact that we see positive regulation of eNOS, a major regulator of angiogenesis, apoptosis, invasion and metastasis, strongly suggests that PDK1-Akt (T308) promotes MPNST growth downstream of erbB4 and thus may be a useful therapeutic target. However, regulation of the PI3K pathway is often further complicated by the expression of multiple PDK1 isoforms that have opposing effects on mTORC1 activity. We would also point out that erbB3, which is also activated by NRG1, contains multiple docking sites for the regulatory p85 subunit of PI3 kinase and thus also likely plays an important role in the regulation of the PI3K/Akt signaling cascade in MPNST cells. It will be of great interest to determine how erbB3 and erbB4 inputs into the PI3K/Akt signaling pathway interact in MPNST cells.

We have also found that erbB4 promotes the phosphorylation of PLC-γ, a protein that promotes the pathogenesis of many tumor types. Inhibition of PLC-γ action in MPNST cells reduced the number of viable cells, indicating that PLC-γ has a similar role in MPNSTs. In addition, we discovered that erbB4 regulates the phosphorylation of two molecules—with-no-lysine kinase 1 (WNK1), a kinase that is phosphorylated at Thr60 by Akt [44], and heat shock protein 60 (HSP60)—that promote tumorigenesis in other cancer types. In glioma cells, WNK1 associates with multiple ion transporters and contributes to migration by modulating volumetric changes [45]. This action is consistent with our observation that *Erbb4* ablation alters the morphology of MPNST cells. However, in HeLa cells, WNK1 associates with mitotic spindles and promotes mitosis and abscission; loss of this kinase results in aberrant mitotic spindle formation, defective cell division and cell death [46]. In contrast to the findings in HeLa cells, we found that WNK1 knockdown did not impair the proliferation and survival of MPNST cells. Further, we commonly observed multinucleated cells in control tumors but not *Erbb4*-null tumors; this is the opposite of what we would expect based on the earlier findings in HeLa cells. These findings thus suggest that WNK1 functions in MPNST cells differ from those observed in other tumor types. Heat shock proteins are upregulated in response to cellular stress and chaperone protein folding. However, heat shock protein expression in tumors is not strictly linked to stress [47]; these proteins also facilitate evasion of apoptosis [48] and help tumor cells escape or inhibit immune system surveillance. These latter HSP60 actions raise the question of whether erbB4 promotes MPNST pathogenesis via analogous mechanisms.

In MPNSTs, we found that erbB4 promotes nuclear signaling by phosphorylating STAT3 and STAT5, two downstream targets of the mTOR and Jak/Stat signaling pathways. Our demonstration that pharmacologic inhibition of STAT3 and STAT5 action decreases the number of viable cells in vitro indicates that phosphorylation of STAT3 and STAT5 is essential for MPNST proliferation and/or survival. This suggestion is consistent with previous observations indicating that elevated expression of both of these STAT proteins is seen in numerous cancers, where their phosphorylation results in enhanced expression of cell cycle progression (e.g., cyclin D1 [49, 50]) and survival (e.g., Bcl-xL and Bcl-2 [51–53]) genes. The absence of STAT5 activation that we observed in *Erbb4*-null cells is consistent with earlier reports that erbB4 activates STAT5, leading to its transport to the nucleus. However, the changes in STAT3 activity were unexpected, as STAT3 is activated by erbB2 or EGFR in other cell types, not erbB4. It is unlikely that erbB2 drives STAT3 phosphorylation in these tumors as *Erbb4*-null MPNST cells remain NRG-1-responsive and express erbB3, a receptor that dimerizes with erbB2 to form an active NRG1 signaling complex. These observations thus raise the interesting possibility that erbB4 regulates STAT3 phosphorylation in MPNST cells via a previously unknown mechanism.

Interestingly, although some signaling cascades were activated in MPNST cells by NRG1β stimulation, others such as p70S6 kinase, STAT3, WNK1 and HSP60 displayed equivalent levels of phosphorylation in the presence and absence of exogenous NRG1β. Nonetheless, *Erbb4* ablation reduced the phosphorylation of these proteins, indicating that erbB4 regulated their phosphorylation. One potential explanation for this conundrum is that the basal level of stimulation provided by tumor cell-derived NRG1 [16] is sufficient to maximally drive the phosphorylation of these proteins. An alternative explanation, however, reflects the fact that erbB4 is not just a NRG1 receptor; it also responds to NRG2, NRG3, NRG4, heparin-binding EGF, betacellulin and epiregulin. We have previously shown that heparin-binding EGF, betacellulin and epiregulin expression is much lower in human MPNST cells than non-neoplastic Schwann cells [27], making it unlikely that these factors play major roles in promoting erbB4 activation. To the best of our knowledge, however, NRG2, NRG3 and NRG4 action has not been carefully examined in MPNSTs. Consequently, we cannot rule out the possibility that one or more of these growth factors contributes to the baseline activation of erbB4 in MPNST cells and drives the phosphorylation of some erbB4 target molecules.

## Conclusions

In summary, multiple erbB4 splice variants are commonly expressed in MPNSTs. Acting in a cell-autonomous manner, these erbB4 variants promote the proliferation and survival of tumor cells. ErbB4 receptors expressed by MPNST cells also indirectly promote angiogenesis in MPNSTs. Despite our anticipation that erbB4 would drive Ras activation, we found that erbB4 instead promotes MPNST pathogenesis via Ras-independent effects; these signaling events include alterations in the PI3K/Akt/mTOR and PLC-γ signaling cascades, activation of transcription via STAT proteins, and the phosphorylation of other molecules with oncogenic potential, such as HSP60. Our findings thus identify erbB4 and key erbB4-dependent signaling pathways as potentially important chemotherapeutic targets in MPNSTs. Future studies will be needed to further investigate the role of the other erbB4 targets that we have identified in MPNST pathogenesis. It will also be of great interest to establish the relative contributions that canonical membrane-based erbB4 signaling and transcriptional signaling by the erbB4 intracellular domain make to the development of these aggressive spindle cell neoplasms.

## Additional file

Additional file 1: **Figure S1.** ErbB4 lysates are sensitive to denaturing detergents. **Table S1.** Patient demographics for immunostained MPNSTs. **Figure S2** Real-time PCR analyses of erbB4 splice variants and mouse MPNST erbB4 expression. **Table S2.** MPNST locations in P_0_-GGFβ3;*Trp53*^+/−^;*Erbb4*^flox/flox^ mice. **Figure S3.** Validation of *Erbb4* ablation and effects on expression of other erbB receptors. **Figure S4.** Kinase array blots for NRG1β and unstimulated control and *Erbb4*-null MPNST cells. **Figure S5.** Downregulation of WNK1 expression did not affect cell viability. (ZIP 7528 kb)

## Data Availability

All data generated or analyzed during this study are included in this published article and supplementary information files.

## Abbreviations

MPNST: Malignant peripheral nerve sheath tumor
NF1: Neurofibromatosis type 1
NRG: Neuregulin
PI3K: Phosphatidylinositol-3 kinase
PLCγ: Phospholipase C γ
RTK: Receptor tyrosine kinase
WNK1: With no lysines 1
