# Supplementary material for: ErbB4 promotes malignant peripheral nerve sheath tumor pathogenesis via Ras-independent mechanisms
Source: Cell Commun Signal. 2019 Jul 10;17:74. doi: 10.1186/s12964-019-0388-5 (PMC6621970; doi:10.1186/s12964-019-0388-5)
Supplement: Supplementary file 1 — Figure S1. ErbB4 lysates are sensitive to denaturing detergents. Table S1. Patient demographics for immunostained MPNSTs. Figure S2 Real-time PCR analyses of erbB4 splice variants and mouse MPNST erbB4 expression. Table S2. MPNST locations in P0-GGFβ3;Trp53+/−;Erbb4flox/flox mice. Figure S3. Validation of Erbb4 ablation and effects on expression of other erbB receptors. Figure S4. Kinase array blots for NRG1β and unstimulated control and Erbb4-null MPNST cells. Figure S5. Downregulation of WNK1 expression did not affect cell viability. (ZIP 7528 kb) [file 12964_2019_388_MOESM1_ESM.zip › reCCS_AdditionalFile_Table_S2.pdf]

Table S2. Locations of tumors isolated from P_0-GGFβ3; *Trp53*$^{+/-}$; *Erbb4*$^{flox/flox}$ mice.

Tumor Location	Frequency
Trigeminal ganglia	55.5%
Dorsal root ganglia	33.5%
Sciatic nerve	5.5%
Probable facial nerve	5.5%
