# Supplementary material for: ErbB4 promotes malignant peripheral nerve sheath tumor pathogenesis via Ras-independent mechanisms
Source: Cell Commun Signal. 2019 Jul 10;17:74. doi: 10.1186/s12964-019-0388-5 (PMC6621970; doi:10.1186/s12964-019-0388-5)
Supplement: Supplementary file 1 — Figure S1. ErbB4 lysates are sensitive to denaturing detergents. Table S1. Patient demographics for immunostained MPNSTs. Figure S2 Real-time PCR analyses of erbB4 splice variants and mouse MPNST erbB4 expression. Table S2. MPNST locations in P0-GGFβ3;Trp53+/−;Erbb4flox/flox mice. Figure S3. Validation of Erbb4 ablation and effects on expression of other erbB receptors. Figure S4. Kinase array blots for NRG1β and unstimulated control and Erbb4-null MPNST cells. Figure S5. Downregulation of WNK1 expression did not affect cell viability. (ZIP 7528 kb) [file 12964_2019_388_MOESM1_ESM.zip › reCCS_AdditionalFile_Table_S1.pdf]

Table S1: Patient demographics for immunostained MPNSTs

Record Number	Diagnosis	Location	Age/race/gender	NF1 Status	erbB4 Score
17	MPNST	Retroperitoneal	24yoBM	yes	4
*3b	MPNST Metastatic	Lung Met	48yoBM	yes	3
*6a	MPNST Grade IV	Chest wall	39yoBM	yes	3
*8a	MPNST Mid Grade	Right arm	61yoWF	NR	3
*10a	MPNST	Left axilla	26yoBF	yes	3
11	MPNST High Grade	Thoracic Spine	54yoWF	NR	3
12	MPNST	Left perisacral	64yoWF	NR	3
15	MPNST	Pleura	76yoM	NR	3
18	MPNST Grade IV	Left buttock	78yoWM	NR	3
19	MPNST Grade IV	Pelvis	33yoBM	yes	3
21	MPNST			NR	3
22	MPNST Mid Grade	Right arm	57yoWF	NR	3
23	MPNST	spine	48yoF	yes	3
4	MPNST with Rhaddomyoblastic differentiation Grad High III	Left neck	42yoM	NR	3
20	MPNST (epitheliod)	Left nasal	57yoWM	NR	3
24	MPNST pleomorphic	Back	71yoWF	NR	3
2	MPNST High Grade	Right forearm	85yoWF	NR	2
*3a	MPNST Mid to High Grade	Left leg	48yoBM	yes	2
*6b	MPNST Grade IV	Pleura	39yoWM	yes	2
*8c	MPNST Metastatic	Lung Met	61yoWF	NR	2
9	MPNST High Grade	Right thigh	27yoBF	yes	2
13	MPNST High Grade Metastatic	Lung Met	73yoWM	NR	2
16	MPNST			NR	2
*1a	MPNST (epitheliod) Grade VI	Left sciatic	42yoWM	NR	1
*14b	MPNST Metastatic	Pleura	30yoWF	yes	1
1b	MPNST (epitheliod) Grade IV	Left thigh/sciatic	42yoWM	NR	0
5	MPNST Mid Grade	Right thigh	72yoWM	NR	0
7	MPNST	extradural	64yoM	NR	0
*8b	MPNST Metastatic	Lung Met	61yoWF	NR	0
*14a	MPNST Grade IV	Lung Met	30yoWF	yes	0
