# Supplementary figures and images for: ErbB4 promotes malignant peripheral nerve sheath tumor pathogenesis via Ras-independent mechanisms
Source: Cell Commun Signal. 2019 Jul 10;17:74. doi: 10.1186/s12964-019-0388-5 (PMC6621970; doi:10.1186/s12964-019-0388-5)

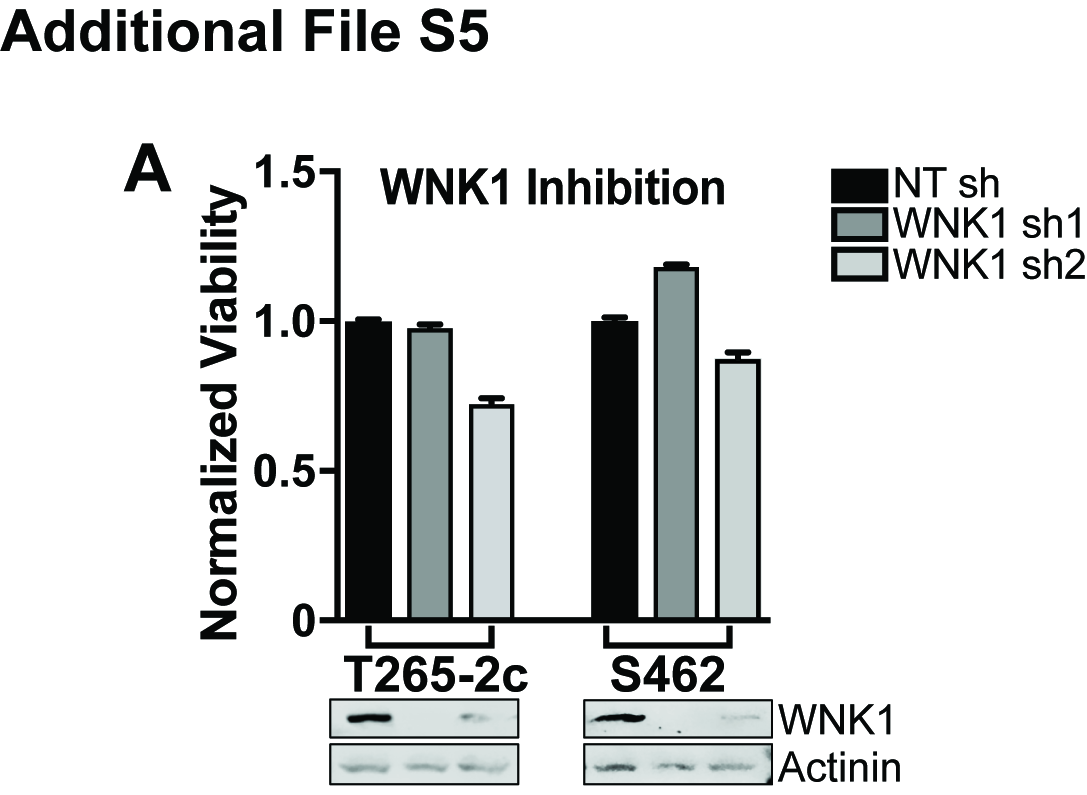

Supplement: Supplementary file 1 — Figure S1. ErbB4 lysates are sensitive to denaturing detergents. Table S1. Patient demographics for immunostained MPNSTs. Figure S2 Real-time PCR analyses of erbB4 splice variants and mouse MPNST erbB4 expression. Table S2. MPNST locations in P0-GGFβ3;Trp53+/−;Erbb4flox/flox mice. Figure S3. Validation of Erbb4 ablation and effects on expression of other erbB receptors. Figure S4. Kinase array blots for NRG1β and unstimulated control and Erbb4-null MPNST cells. Figure S5. Downregulation of WNK1 expression did not affect cell viability. (ZIP 7528 kb) [file 12964_2019_388_MOESM1_ESM.zip › reCCS_AdditionalFile_S5.tif]

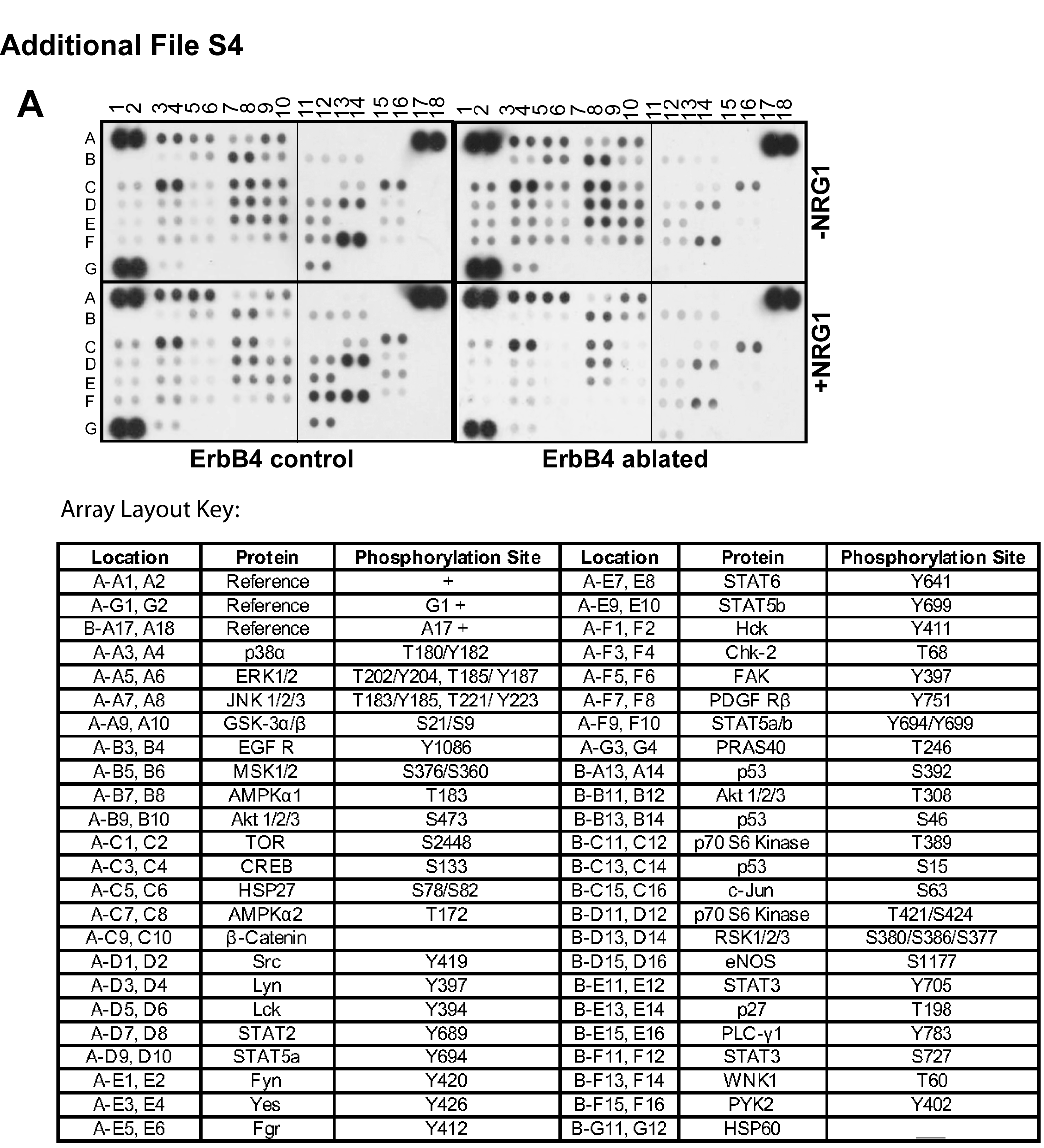

Supplement: Supplementary file 1 — Figure S1. ErbB4 lysates are sensitive to denaturing detergents. Table S1. Patient demographics for immunostained MPNSTs. Figure S2 Real-time PCR analyses of erbB4 splice variants and mouse MPNST erbB4 expression. Table S2. MPNST locations in P0-GGFβ3;Trp53+/−;Erbb4flox/flox mice. Figure S3. Validation of Erbb4 ablation and effects on expression of other erbB receptors. Figure S4. Kinase array blots for NRG1β and unstimulated control and Erbb4-null MPNST cells. Figure S5. Downregulation of WNK1 expression did not affect cell viability. (ZIP 7528 kb) [file 12964_2019_388_MOESM1_ESM.zip › reCCS_AdditionalFile_S4.tif]

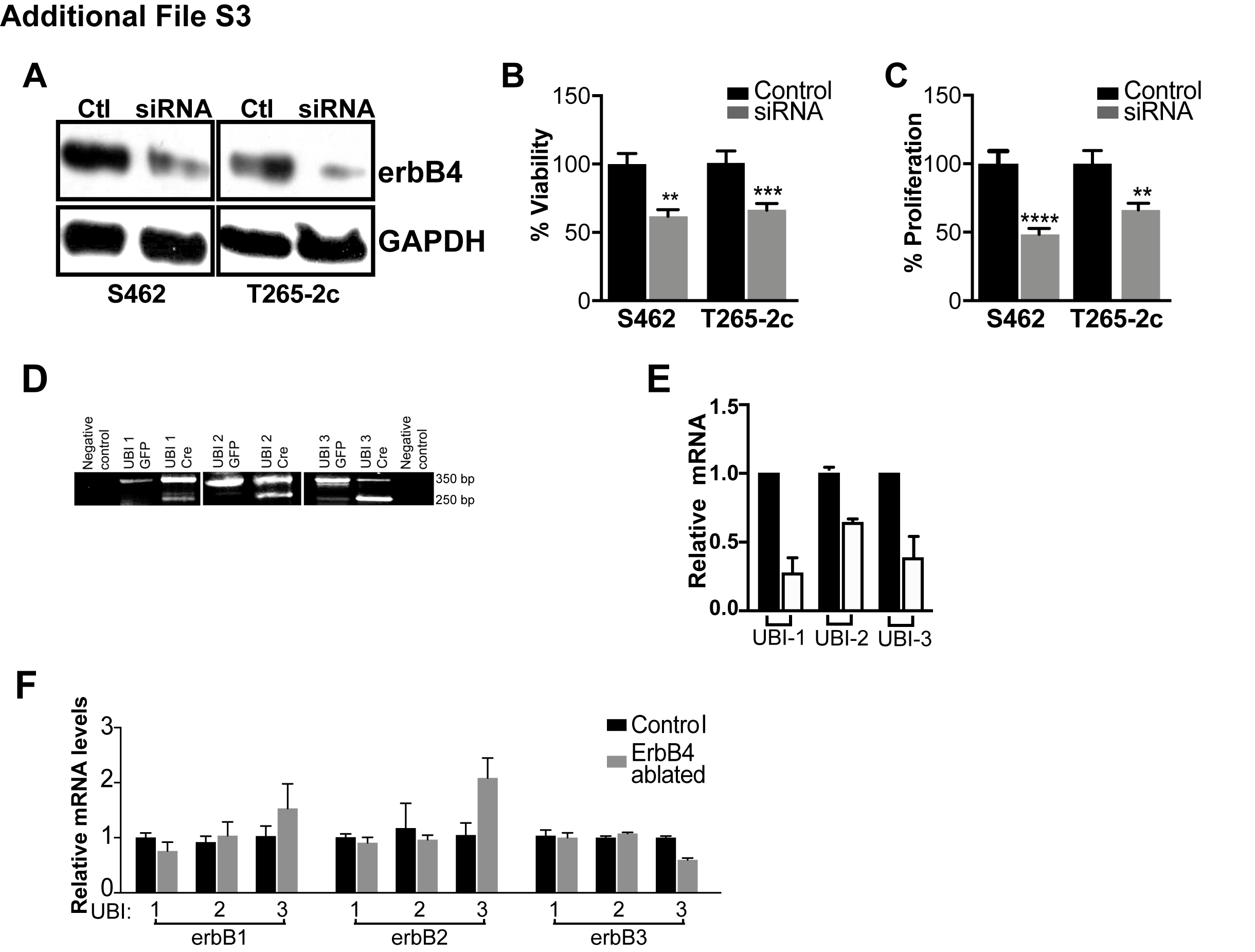

Supplement: Supplementary file 1 — Figure S1. ErbB4 lysates are sensitive to denaturing detergents. Table S1. Patient demographics for immunostained MPNSTs. Figure S2 Real-time PCR analyses of erbB4 splice variants and mouse MPNST erbB4 expression. Table S2. MPNST locations in P0-GGFβ3;Trp53+/−;Erbb4flox/flox mice. Figure S3. Validation of Erbb4 ablation and effects on expression of other erbB receptors. Figure S4. Kinase array blots for NRG1β and unstimulated control and Erbb4-null MPNST cells. Figure S5. Downregulation of WNK1 expression did not affect cell viability. (ZIP 7528 kb) [file 12964_2019_388_MOESM1_ESM.zip › reCCS_AdditionalFile_S3.tif]

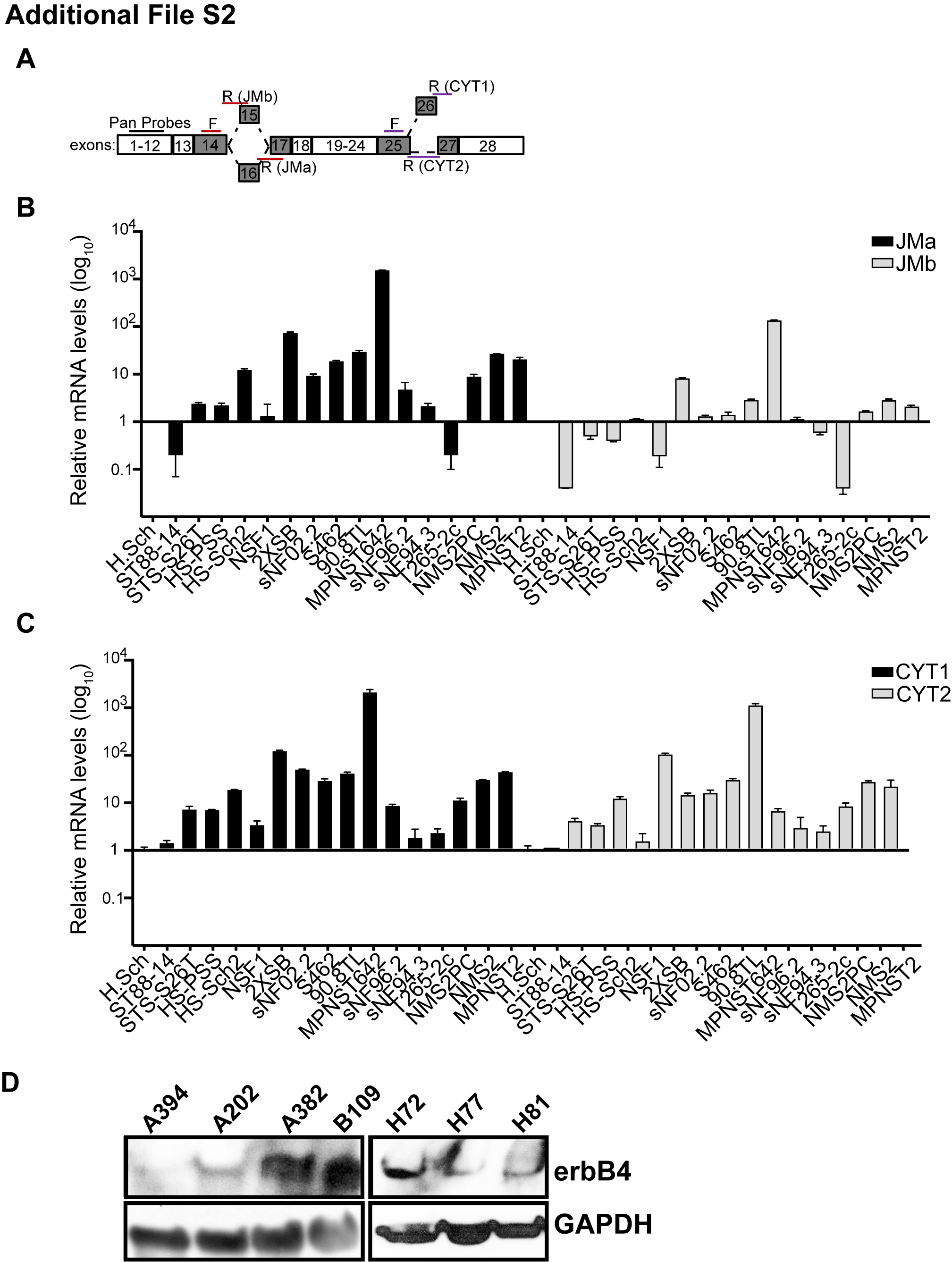

Supplement: Supplementary file 1 — Figure S1. ErbB4 lysates are sensitive to denaturing detergents. Table S1. Patient demographics for immunostained MPNSTs. Figure S2 Real-time PCR analyses of erbB4 splice variants and mouse MPNST erbB4 expression. Table S2. MPNST locations in P0-GGFβ3;Trp53+/−;Erbb4flox/flox mice. Figure S3. Validation of Erbb4 ablation and effects on expression of other erbB receptors. Figure S4. Kinase array blots for NRG1β and unstimulated control and Erbb4-null MPNST cells. Figure S5. Downregulation of WNK1 expression did not affect cell viability. (ZIP 7528 kb) [file 12964_2019_388_MOESM1_ESM.zip › reCCS_AdditionalFile_S2.tif]

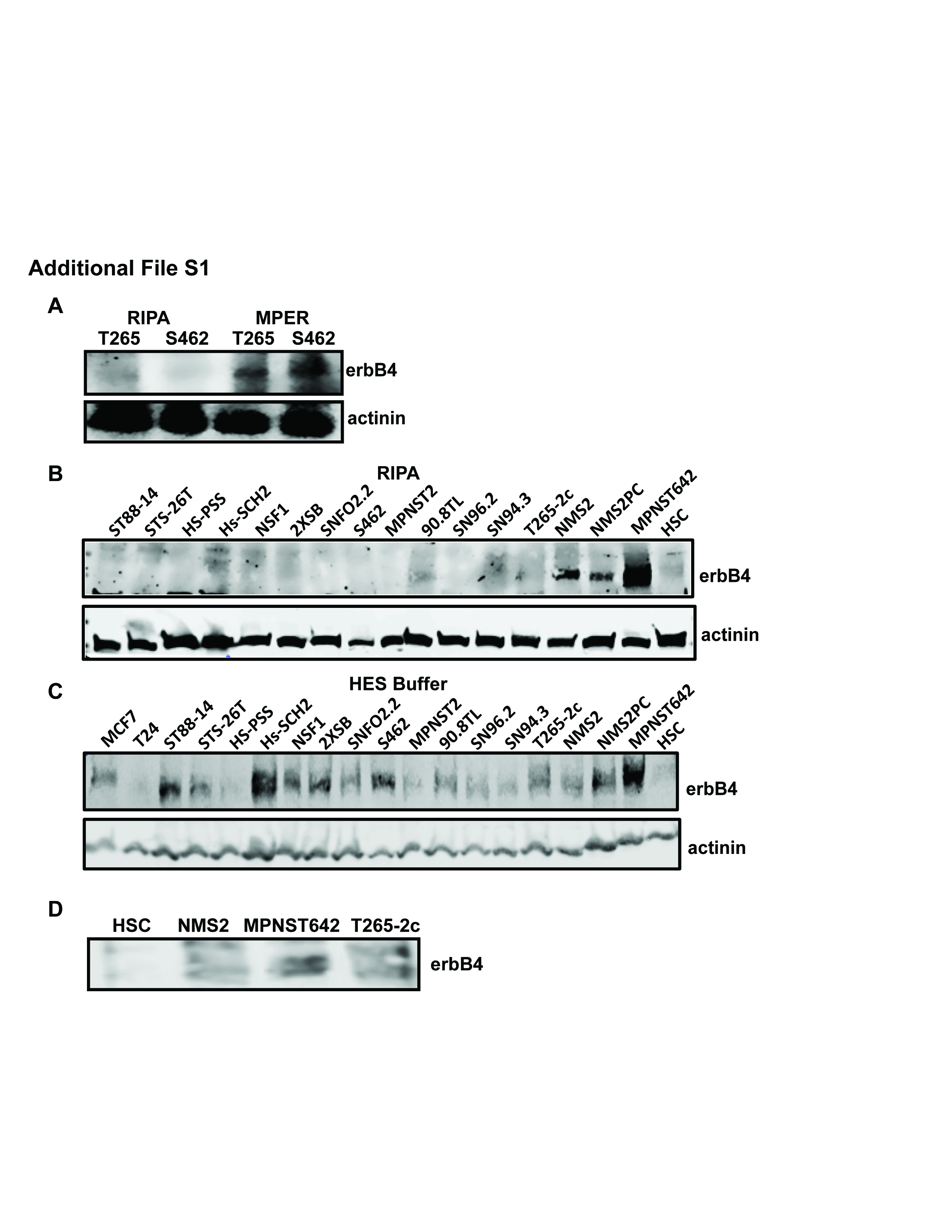

Supplement: Supplementary file 1 — Figure S1. ErbB4 lysates are sensitive to denaturing detergents. Table S1. Patient demographics for immunostained MPNSTs. Figure S2 Real-time PCR analyses of erbB4 splice variants and mouse MPNST erbB4 expression. Table S2. MPNST locations in P0-GGFβ3;Trp53+/−;Erbb4flox/flox mice. Figure S3. Validation of Erbb4 ablation and effects on expression of other erbB receptors. Figure S4. Kinase array blots for NRG1β and unstimulated control and Erbb4-null MPNST cells. Figure S5. Downregulation of WNK1 expression did not affect cell viability. (ZIP 7528 kb) [file 12964_2019_388_MOESM1_ESM.zip › reCCS_AdditionalFile_S1.tif]
